# Patient Perspectives on Approval Speed vs Evidentiary Certainty in US Cancer Drug Approvals

**DOI:** 10.1001/jamanetworkopen.2026.17450

**Published:** 2026-06-09

**Authors:** Robin Forrest, Ajay Aggarwal, Michelle Tregear, Melanie Wyne, Holly Fernandez Lynch, Anita K. Wagner, Emily Jackson, Huseyin Naci

**Affiliations:** 1 Department of Health Policy, London School of Economics and Political Science, London, United Kingdom; 2 Department of Health Services Research and Policy, London School of Hygiene and Tropical Medicine, Department of Oncology, Guy’s and St Thomas’ NHS Trust, London, United Kingdom; 3 National Breast Cancer Coalition, Washington, DC; 4 Department of Medical Ethics and Health Policy, The Perelman School of Medicine, Carey Law School, University of Pennsylvania, Philadelphia; 5 Department of Population Medicine, Harvard Medical School and Harvard Pilgrim Health Care Institute, Boston, Massachusetts; 6 Law School, London School of Economics and Political Science, London, United Kingdom

## Abstract

**Importance:**

Use of the US Food and Drug Administration’s Accelerated Approval pathway assumes that patients are willing to accept uncertainty about clinical benefit in exchange for faster access to new cancer drugs. However, little is known about how patients view this trade-off or the circumstances under which they consider it acceptable.

**Objectives:**

To explore patients’ views on the trade-off between faster approval and evidentiary certainty regarding the clinical benefit of new cancer drugs in the US.

**Design, Setting, and Participants:**

This qualitative study used semistructured interviews conducted online between January 27 and April 1, 2025, with patients aged 18 years or older diagnosed with breast cancer. Purposive sampling was used to achieve maximum variation in cancer stage, treatment status, age, and sociodemographic background.

**Main Outcomes and Measures:**

The primary outcome was patients’ views on the trade-off between faster drug approval and evidentiary certainty. Interview transcripts were thematically analyzed to identify key patterns in understanding, experiences of uncertainty, attitudes toward waiting, treatment priorities, and views on regulatory decision-making.

**Results:**

A total of 125 individuals registered their interest and met eligibility criteria to participate. After purposive sampling, 30 participated in this study (10 aged 20-39 years [33.3%], 12 aged 40-59 years [40.0%], and 8 aged >60 years [26.7%]; all female), representing 17 US states (9 Northeast, 12 Midwest, 4 South, 5 West). A total of 13 participants (43.3%) had metastatic disease, 21 (70.0%) were receiving active treatment, and 27 (90.0%) had received at least 1 systemic therapy. Faster approval at the expense of certainty about the clinical benefit of new cancer drugs was considered most acceptable when no alternative treatments existed or when the anticipated benefits were transformative. When clinical benefit was uncertain, participants emphasized the importance of survival and quality of life as priority treatment outcomes, the added risks of adverse effects, and the burden of intensive treatment. Many viewed broader access to clinical trials, rather than faster regulatory approval, as an effective way to address unmet needs while facilitating evidence generation for approval.

**Conclusion and Relevance:**

In this qualitative study of patients diagnosed with breast cancer, there was a mismatch between when patients considered trading evidentiary certainty for faster approval as acceptable and the conditions under which many new cancer drugs are approved by the Food and Drug Administration. To better align regulatory approvals with patient values, use of accelerated approval may be most appropriate for drugs that are likely to address genuine treatment gaps or offer meaningful improvements in clinical outcomes over existing alternatives.

## Key Points

**Question:**

What are patients’ views on the trade-off between faster drug approval and evidentiary certainty, and when is this most acceptable?

**Findings:**

In this qualitative study interviewing 30 patients with breast cancer, participants expressed that high uncertainty about the clinical benefit of a new cancer drug at approval was most acceptable if no alternative treatment options existed or if the anticipated benefit of a new drug was transformative.

**Meaning:**

These findings suggest that patients’ willingness to accept evidentiary uncertainty for faster cancer drug approval does not align with the conditions under which most new cancer drugs are currently approved.

## Teaser

This qualitative study explores patients’ views on the trade-off between faster US Food and Drug Administration approval and evidentiary certainty regarding clinical benefits of new cancer drugs.

## Introduction

The US Food and Drug Administration (FDA) is tasked by law with ensuring that new drugs are safe and effective.^1^ In fulfilling this role, the agency must balance timely approval with evidentiary certainty needed to demonstrate clinical benefit. Because meaningful clinical benefit (eg, overall survival benefit) can take years to establish,^2^ approval decisions often involve a trade-off between earlier approval and evidentiary certainty.

Debate over this evidence vs access trade-off has focused on the FDA’s Accelerated Approval pathway, which facilitates earlier drug approval for serious conditions using surrogate end points “reasonably likely”^3,4^ to predict clinical benefit in how a patient feels, functions, or survives. After approval, sponsors must conduct confirmatory trials to verify clinical benefit, but in the interim, patients face uncertainty about whether a drug would benefit them.^5^ The FDA justifies accelerated approval on the assumption that patients and caregivers are willing “to accept less certainty about effectiveness in exchange for earlier access to much needed medicines.”^6^ Although plausible, this assumption has not been fully examined. Emerging research has suggested that some individuals with experience of cancer may be willing to wait for approval of new cancer drugs in exchange for greater certainty of survival benefit, depending on prognosis, age, and other factors.^7^

Breast cancer provides a critical context in which to examine this trade-off. Approximately 1 in 8 women in the US will be diagnosed with breast cancer in their lifetime, with outcomes and unmet needs varying widely by disease stage, subtype, and sociodemographic characteristics.^8,9^ These differences may shape how patients weigh earlier approval against evidentiary certainty. While some new breast cancer drugs have substantially improved clinical outcomes for patients,^10,11^ this is not always the case. Over the past 2 decades, the FDA approved 42 indications across 24 drugs for breast cancer; 41% have demonstrated an improvement in overall survival (median, 2.8 months), and 14% have demonstrated a quality-of-life benefit.^12^ Eight indications were granted accelerated approval, including 2 later withdrawn due to lack of confirmed clinical benefit (bevacizumab in 2011 and atezolizumab in 2021). Similar uncertainty has also been documented in other tumor types.^13,14^

Although the evidence vs access trade-off directly affects patients, no study has qualitatively examined their views. We therefore conducted semistructured interviews with a diverse group of patients with breast cancer in the US to explore their understanding of FDA drug approval, attitudes toward this trade-off, perceptions of whether the FDA achieves the right balance, and priorities for improvement.

## Methods

This qualitative study was approved by the London School of Economics Research Ethics Committee. All participants provided verbal informed consent and were reimbursed with a $50 gift card. The study followed the Consolidated Criteria for Reporting Qualitative Research (COREQ) reporting guideline.^15^

### Setting, Population, and Recruitment

Study recruitment was facilitated by the US National Breast Cancer Coalition, which distributed a study advertisement via email to 3 national-level and 7 state-level breast cancer support organizations represented on its national board. These organizations were selected for the clinical and sociodemographic diversity of their constituents (eFigure in Supplement 1). To be eligible, participants must have been diagnosed with breast cancer, aged 18 years or older, and living in the US. No exclusion criteria were applied. Eligible individuals completed an online registration form to provide key clinical and demographic information, which formed the sampling frame. Purposive sampling was applied to achieve maximum variation in cancer stage, treatment status, age, self-reported sex (female, male, intersex, prefer not to say), self-reported race and ethnicity (American Indian, Native American, or Alaska Native; Asian or Asian American; Black or African American; Hispanic or Latino; Middle Eastern, North African, or Mediterranean; Native Hawaiian or Pacific Islander; White or European American; or other [free text]) and sociodemographic background identified a priori as factors likely to shape views on uncertainty.^7^ The race and ethnicity categories were selected to reflect the racial and ethnic groups most commonly represented in the US breast cancer population. Sampling continued until diversity across these characteristics and theoretical saturation (ie, no new themes or insights emerged from additional interviews) were achieved.

### Interview Guide Design

The interview guide was developed collaboratively by the interdisciplinary author team. Two patient participants also took part in a 90-minute pilot session, providing feedback on recruitment, interview format, question relevance, comprehension, and burden. Further pilot testing was conducted with 5 lay individuals. The final semistructured guide comprised 7 main questions with subquestions and prompts covering understanding of FDA approval, attitudes toward the evidence vs access trade-off, whether the FDA achieves the right balance, and priorities for improvement (eTable 1 in Supplement 1).

### Data Analysis

All interviews were conducted by 1 researcher with training in qualitative research (R.F.) between January 27 and April 1, 2025. Interviews were digitally recorded, transcribed verbatim, deidentified, and thematically coded by R.F. using ATLAS.ti, version 25.0.1 (ATLAS.ti Scientific Software Development GmbH). Thematic analysis followed the Braun and Clarke 6-phase process.^16^ A preliminary coding frame was developed during pilot testing and iteratively refined as data collection progressed. Both deductive and inductive coding were used to identify manifest content (eg, understanding) and latent content (eg, values, reasoning) related to the research questions. After 5 interviews, coding was cross checked by H.N. to ensure that the breadth and depth of relevant concepts were captured. All authors contributed to the development of key themes through multiple meetings.

## Results

### Recruitment and Participants

A total of 125 individuals registered their interest and met eligibility criteria. Using purposive sampling, 45 individuals were contacted, of whom 30 (66.7%) participated in this study (10 aged 20-39 years [33.3%], 12 aged 40-59 years [40.0%], and 8 aged ≥60 years [26.7%]; all identifying as female; 4 identifying as Asian or Asian American [13.3%], 4 as Black or African American [13.3%], 2 as Hispanic or Latino [6.7%], and 20 as White or European American [66.7%]), at which point thematic saturation was reached (Table 1). Of the 15 individuals who did not participate, 14 (93.3%) did not respond to the invitation, and 1 (6.7%) was no longer able to participate due to receiving treatment. Interviews were conducted online (audio and video) and lasted a mean (SD) of 53 (13) minutes. Participants were from 17 US states (9 Northeast, 12 Midwest, 4 South, and 5 West). At the time of their interview, 21 participants (70%) were receiving active treatment, 13 (43.3%) had metastatic disease, and 27 (90.0%) had received at least 1 systemic therapy. Most participants (27 [90.0%]) reported no prior training in drug approval, and 28 (93.3%) reported no relevant organizational affiliations. Individual participant characteristics are provided in eTable 2 in Supplement 1.

**Table 1.** Participant Characteristics (N = 30)

Characteristic	Participants, No. (%)
Sex	
Female	30 (100)
Male	0
Intersex	0
Prefer not to say	0
Age, y	
20-29	2 (6.7)
30-39	8 (26.7)
40-49	5 (16.7)
50-59	7 (23.3)
60-69	7 (23.3)
70-79	1 (3.3)
Race and ethnicity	
Asian or Asian American	4 (13.3)
Black or African American	4 (13.3)
Hispanic or Latino	2 (6.7)
White or European American	20 (66.7)
Other^a^	0
Education	
College degree	10 (33.3)
Graduate degree	9 (30.0)
Some college	8 (26.7)
High school diploma or equivalent	3 (10.0)
US states represented, No.	17
Household income, $	
<20 000	2 (6.7)
20 000-39 999	4 (13.3)
40 000-59 999	3 (10.0)
60 000-79 999	3 (10.0)
80 000-99 999	6 (20.0)
≥100 000	7 (23.3)
Preferred not say	5 (16.7)
Health coverage^b^	
Employer	20 (66.7)
Medicaid	5 (16.7)
Medicare	2 (6.7)
Nongroup or individual	3 (10.0)
No health insurance or uninsured	2 (6.7)
Military or Veterans Administration	1 (3.3)
Time since diagnosis, median (IQR) [range], y	4.5 (2.0-8.8) [1-19]
Active treatment	
Yes	21 (70.0)
No	9 (30.0)
Breast cancer stage (at interview)	
No evidence of disease	5 (16.7)
Early stage (I or II)	5 (16.7)
Locally advanced (III)	7 (23.3)
Metastatic or advanced (IV)	13 (43)
Treatment types received	
Surgery	23 (76.7)
Radiotherapy	21 (70.0)
Chemotherapy	27 (90.0)
Hormone therapy	19 (63.3)
Targeted or immunotherapy	18 (60.0)
Self-reported understanding of FDA approval	
1 (No knowledge)	4 (13.3)
2	9 (30.0)
3	12 (40.0)
4	5 (16.7)
5 (Expert knowledge)	0
Training in drug approval	
No	27 (90.0)
Yes (as a psychologist)	1 (3.3)
Yes (at a conference or virtual webinar)	1 (3.3)
Yes (during a clinical trial)	1 (3.3)
Affiliations	
No	28 (93.3)
Yes (previously employed as a pharmacist)	2 (6.7)

Abbreviation: FDA, US Food and Drug Administration.

^a^ Included American Indian, Native American, or Alaska Native; Middle Eastern, North African, or Mediterranean; Native Hawaiian or Pacific Islander; or other (free text).

^b^ Some participants reported more than 1 type of health coverage.

### Themes

Six overarching themes (each with several subthemes) emerged from the analysis. The Figure provides an overview of these themes, and illustrative quotes are provided in the tables. Additional supporting quotes for each subtheme are provided in eTable 3 in Supplement 1.

**Figure.**
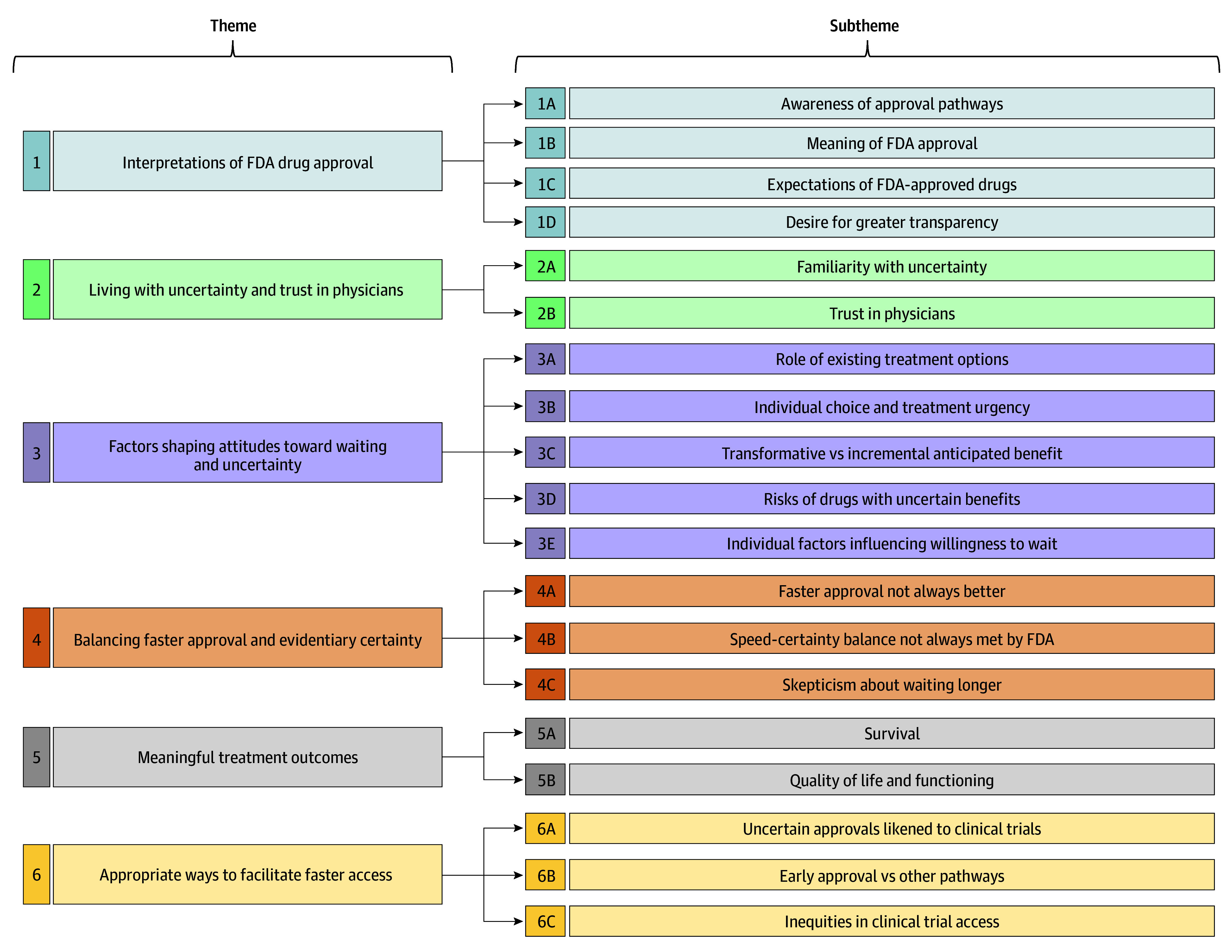
Flow Diagram Overviewing the Key Themes and Subthemes That Shaped Patients’ Views on the Trade-Off Between Faster Drug Approval and Evidentiary Certainty Regarding the Clinical Benefit of New Cancer Drugs FDA indicates US Food and Drug Administration.

#### Theme 1: Interpretations of FDA Drug Approval

Almost all participants knew that the FDA was responsible for approving new cancer drugs in the US, but few were aware of different approval pathways. Approval was interpreted in different ways. For many participants, it meant that a drug’s benefits outweigh its risks or that a drug was “safe and effective”; other participants assumed it meant that a drug “helps most people,” or was “better than the standard of care.” Expectations of approved drugs were also mixed. Some participants expressed optimism, expecting a “good success rate” or “lesser side effects” compared with other approved drugs, whereas others were more skeptical, with 1 participant saying that “the only surefire thing is that I will never get cured,” and another adding that approval did not mean a drug would be right for them. Several participants highlighted the need for greater transparency, calling for clearer information on “the good, the bad, and the ugly” of new drugs, including “who [the drug] did help, and who it did not help” and “why [the FDA] made this determination.” Supportive quotes for theme 1 are provided in Table 2.

**Table 2.** Illustrative Quotations for Themes 1 and 2

Theme and subthemes	Illustrative quotes (participant identifier, age, cancer stage)^a^
**1. Interpretations of FDA drug approval**	
1A. Awareness of approval pathways	“I cannot say with certainty that I know that there are different [approval pathways], but I imagine the approval process for chemotherapy is different than the approval process for immunotherapy.” (P6, 30-39 y, early stage) “I have no idea, but I would actually guess that they’re different.” (P10, 30-39 y, metastatic) “I think that [all approval pathways are] the same for cancer drugs.” (P24, 50-59 y, metastatic)
1B. Meaning of FDA approval	“It means that there’s enough science to back it up to where I know that it is safe and effective.” (P8, 20-29 y, locally advanced) “I would imagine that [approved drugs] are either meeting the standard of care or they’re better than the standard of care.” (P10, 30-39 y, metastatic) “Approved means that based on the trials and the outcomes that they see, that the drug overall helps most people.” (P30, 60-69 y, NED)
1C. Expectations of FDA-approved drugs	“You can’t really have expectations because there’s so much variety; there’s like, not a surefire thing, right? The only surefire thing is that I will never get cured.” (P3, 40-49 y, metastatic) “I still do not believe that every drug is for me. Okay, [irrespective of] the fact that they’ve been approved.” (P14, 30-39 y, locally advanced) “Even if it’s approved, it’s going to come with side effects…[but] I would expect lesser side effects than already on the market cancer drugs.” (P21, 30-39 y, locally advanced)
1D. Desire for greater transparency	“I think just [the FDA] could make the process a little bit easier for laypeople to understand…how it was studied…how many people in the studies, these were the concerns, and this is why we made this determination.” (P7, 30-39 y, early stage) “I think it’s good whenever [the FDA] put out, like articles or anything like that…like, oh, here was the significance, and who it did it help? Who did it not help?…explaining both sides.” (P18, 20-29 y, locally advanced) “[I want] the good, the bad, and the ugly...I want it all. I don’t want just the good…I want all the information. That way I can determine what’s best for me.” (P22, 50-59 y, metastatic)
**2. Living with uncertainty and trust in physicians**	
2A. Familiarity with uncertainty	“I think it’s just really a crapshoot...it might work for me. It might not work for me.” (P4, 60-69 y, early stage) “And my doctor, they’re like…let’s try it, and if it doesn’t work…we can switch you to something else. You know, I have heard that a lot through my diagnosis too, which is yucky.” (P29, 60-69 y, NED) “A lot of times with these cancer drugs, they can’t guarantee…your body is different than mine…the side effects…it’s not a one size fits all…there’s no guarantees.” (P5, 30-39 y, locally advanced)
2B. Trust in physicians	“My oncologist has been working with cancer and cancer patients and research 20 plus years. It gives you a little bit of comfort that maybe he knows what he’s talking about.” (P1, 40-49 y, metastatic) “Personally with my oncologist, I would feel very comfortable with whatever she recommended.” (P12, 50-59 y, metastatic) “If you [my doctor] are confident, then I will be confident, because that’s your wheelhouse.” (P10, 30-39 y, metastatic)

Abbreviations: FDA, US Food and Drug Administration; NED, no evidence of disease.

^a^ Additional quotes in support of themes 1 and 2 are provided in eTable 3 of Supplement 1.

#### Theme 2: Living With Uncertainty and Trust in Physicians

Participants commonly described uncertainty as an everyday feature of their cancer care. Drug treatment often felt like a “crapshoot” that involved trial and error, with even well-established drugs providing “no guarantees.” To navigate this uncertainty, participants relied on their confidence and trust in their physicians’ expertise and experience. Supportive quotes for theme 2 are provided in Table 2.

#### Theme 3: Factors Shaping Attitudes Toward Waiting and Uncertainty

Participants identified several key factors that shaped their attitudes toward waiting for new drugs and accepting uncertainty. Foremost was the availability and adequacy of existing treatment options. When facing a “last resort,” or having “run out of options,” uncertainty was preferable to having no treatment at all. By contrast, when alternative treatments were available, participants emphasized the need for greater certainty, of whom one said, “If there’s a drug that already treats that symptom or that cancer that we know is safe and effective, there’s no reason to speed up the approval process.” Two consistent underlying considerations were the importance of individual choice and urgency to treat cancer before progression. As 1 participant stated, “Anyone who is facing…death, gets to choose whether they want to take something new and revolutionary that doesn’t necessarily have as many studies as they want,” while another added, “With cancer, you don’t really have much time to wait.”

The anticipated benefit of a drug also strongly influenced views. Potentially transformative drugs, described as those that “guaranteed, like 10 more years, 20 more years,” “dramatically improve[d] outcomes,” or were “a breakthrough,” made participants more willing to accept uncertainty. In contrast, drugs offering “similar results and similar side effects” or competing with existing treatments were viewed as less justifiable for early approval.

Additional risk of harm was a key consideration when potential benefits were uncertain, particularly on top of already-extensive treatment regimens. As 1 participant explained, “With cancer, you’ve already experienced enough harm…you don’t want to take anything that is going to be a detriment to your quality of life,” and another adding, “You don’t want to cause more damage than what is already there.” Finally, personal circumstances, including age, cancer stage, quality of life, and prognosis, also shaped some participants’ attitudes about waiting and uncertainty. Supportive quotes for theme 3 are provided in Table 3.

**Table 3.** Illustrative Quotations for Themes 3 and 4

Theme and subthemes	Illustrative quote (participant identifier, age, cancer stage)^a^
**3. Factors shaping attitudes toward waiting and uncertainty**	
3A. Role of existing treatment options	“When it’s the last resort medication…[some patients] would rather try something than not have a chance at it at all.” (P1, 40-49 y, metastatic) “If there’s a drug that already treats that symptom or that cancer that we know is safe and effective, then there’s no reason to speed up the approval process.” (P6, 30-39 y, early stage) “If there are other drugs available for that particular type of cancer that work, then I think [the FDA] needs to be certain.” (P16, 50-59 y, NED) “If there are no other alternatives, I think something is better than nothing…I’d much rather deal with that uncertainty.” (P18, 20-29 y, locally advanced) “I think if I had run out of options, then I would be okay with [more uncertainty].” (P24, 50-59 y, metastatic)
3B. Individual choice and treatment urgency	“Anyone who is facing…death, gets to choose whether they want to take something new…that doesn’t have as many studies as they want.” (P23, 40-49 y, NED) “I’m not going to sit around for 6 months and wait for more data to come out.” (P4, 60-69 y, early stage) “With cancer, you don’t really have much time to wait.” (P18, 20-29 y, locally advanced)
3C. Transformative vs incremental anticipated benefit	“If there was a new drug that’s going to compete with 4 other potential chemotherapies for early-stage breast cancer, I might say, hold off unless it was showing very minimal side effects or if it could be used in people who can’t do the other types of chemotherapies…[otherwise] there’s no reason to speed it up.” (P6, 30-39 y, early stage) “If it’s really going to be a breakthrough, we should push harder to get it approved….if [it] guaranteed, like, 10 more years, 20 more years.” (P20, 50-59 y, metastatic) “[If it is] working on every single person, and [they] lived for 20 more years, and it’s guaranteed, then sure, push it through.” (P10, 30-39 y, metastatic)
3D. Risks of drugs with uncertain benefits	“With cancer, you’ve experienced enough harm…you don’t want to take anything that is going to be a detriment to your quality of life.” (P9, 40-49 y, early stage) “You don’t want to cause more damage than what is already there.” (P15, 30-39 y, locally advanced) “I would probably…focus on the safety of what we’re approving and not the speed.” (P29, 60-69 y, NED)
3E. Individual factors influencing willingness to wait	“If you’re asking an 80-y-old person, I don’t think I would put myself through [certain treatments]…whereas, at [age in her 30s], I hopefully have only half my life done…I’m willing to put myself through a lot.” (P5, 30-39 y, locally advanced) “Because I’ve had no evidence of disease for almost 4 years, I personally would be more hesitant to try something, but I know that people with the more advanced stage [cancer]…they would absolutely be more willing to try.” (P12, 50-59 y, metastatic) “I hate to say this, but maybe at stage IV, when you’re kind of at the end of the line, [patients] would be more accepting of a new drug that is has uncertainty around it.” (P21, 30-39 y, locally advanced)
**4. Balancing faster approval and evidentiary certainty**	
4A. Faster approval not always better	“No, [faster approval] it’s not always better.” (P4, 60-69 y, early stage) “I probably feel in the very middle of the 2 extremes…a lot of people would think, no, faster, earlier is more dangerous…[but others] think, faster and earlier is basically getting in early. So I think probably there’s a 50/50, split.” (P18, 20-29 y, locally advanced)
4B. Speed-certainty balance not always met by FDA	“I think that there’s a lot of things that could be faster out there…I’m sure it’s a bureaucracy” (P5, 30-39 y, locally advanced) “I actually think that the FDA takes too long. I think it could do it quicker.” (P7, 30-39 y, early stage) “I think there are times that [the FDA] put a drug on the market too soon, without knowing enough information.” (P12, 50-59 y, metastatic) “I think that [the FDA is] pushed…that the drug companies have a lot of influence.” (P17, 50-59 y, metastatic)
4C. Skepticism about waiting longer	“[Waiting longer for greater certainty that a cancer drug works before approval] is a fallacy…there is no certain...nothing is certain.” (P1, 40-49 y, metastatic) “When you have these time bombs in your body that are trying to kill you, time matters. So I’m not quite sure it’s a good thing to for them to wait.” (P27, 60-69 y, metastatic) “I guess I need to understand what the definition of certainty is…you know, because, like we discussed earlier, nothing is 100%, so you should never be waiting for 100% because we’re just not there in terms of cancer treatment.” (P28, 60-69 y, metastatic)

Abbreviations: FDA, US Food and Drug Administration; NED, no evidence of disease.

^a^ Additional quotes in support of themes 3 to 4 are provided in eTable 3 of Supplement 1.

#### Theme 4: Balancing Faster Approval and Evidentiary Certainty

Many participants felt that faster drug approval could be appropriate “in some circumstances” but stressed that it was “not always better.” Views on whether the FDA achieves the right balance were mixed. Some believed that the FDA was too slow and hindered by bureaucracy, while others believed it was too quick to approve new drugs, perhaps influenced by drug companies. Several participants questioned whether certainty was achievable at all, describing it as a “fallacy” and insisting that “nothing is certain” and that “you should never be waiting for 100% because we’re just not there in terms of cancer treatment.” Supportive quotes for theme 4 are provided in Table 3.

#### Theme 5: Meaningful Treatment Outcomes

Participants consistently identified overall survival and quality of life as the most important treatment outcomes. Several felt that FDA approvals should be based on evidence of these outcomes, even if this takes additional time, with 1 participant saying, “You do have to see if [a new drug] is going to extend life, and that takes time. There’s no way that you can step around that.” Participants also emphasized that improved survival was only meaningful if accompanied by preserved quality of life and functional status. As 1 participant put it, “Am I still able to walk? Am I still able to eat, able to dress myself, able to move around independently…those are pretty important to me.” Another participant added, “You can keep me alive for another 6 years, but if I can’t get on the ground and play with my grandchildren, or I can’t, you know, go for a walk with my husband, it’s really not worth it.” Supportive quotes for theme 5 are provided in Table 4.

**Table 4.** Illustrative Quotations for Themes 5 and 6

Theme and subthemes	Illustrative quote (participant identifier, age, cancer stage)^a^
**5. Meaningful treatment outcomes**	
5A. Survival	“Time is very precious.” (P8, 20-29 y, locally advanced) “You do have to see if [a new drug is] going to extend life, and that takes time. There’s no way that you can step around that…and then the quality of life while it’s extending the time…those are the things that really matter at the end of the day.” (P28, 60-69 y, metastatic)
5B. Quality of life and functioning	“I would be okay with [some uncertainty of clinical benefit], as long as [the drug] doesn’t affect like activities of daily living. Like, am I still able to walk…eat…dress myself…move around independently. I think those are pretty important to me.” (P11, 50-59 y, metastatic) “Quality of life is better than quantity of life, and that’s what I have chosen [in my treatment]…but they both kind of go hand in hand.” (P17, 50-59 y, metastatic) “My understanding is there is no cure at this moment. And so my objective with certain medications is stability…quality of life…functioning.” (P3, 40-49 y, metastatic)
**6. Appropriate ways to facilitate faster access**	
6A. Uncertain approvals likened to clinical trials	“Well, I mean, that [approval without certainty of clinical benefit] to me, is a clinical trial…to me that’s more like testing.” (P4, 60-69 y, early stage) “Isn’t that? Wouldn’t that be part of a clinical trial?” (P28, 60-69 y, metastatic)
6B. Early approval vs other pathways	“The FDA shouldn’t approve something for general public, but if something is available and a patient is willing to [try]…then the patient should have access...having patient access vs having FDA approval do different things.” (P2, 40-49 y, NED) “Maybe there’s a way that these people can be given the drug without it being approved by the FDA...in a more systematic way, with the understanding that we don’t have anything else available.” (P7, 30-39 y, early stage)
6C. Inequities in clinical trial access	“Some [patients can access clinical trials], but not all of them…[if] your hospital or clinic doesn’t offer it, you might not be able to get to it. You might have to commit to travel, and that’s not feasible for everybody.” (P10, 30-39 y, metastatic) “I think there should be more clinical trials available to different demographics of people, and that way there are more test subjects there…more accuracy or more numbers of how this drug affects different people, and if it’s doing its job.” (P15, 30-39 y, locally advanced) “Early access could be good in the sense if you’re contributing to the research pool…and that helps build the approval along, sure, right, that’s great.” (P28, 60-69 y, metastatic)

Abbreviations: FDA, US Food and Drug Administration; NED, no evidence of disease.

^a^ Additional quotes in support of themes 5 and 6 are provided in eTable 3 of Supplement 1.

#### Theme 6: Appropriate Ways to Facilitate Faster Access

Participants offered broader perspectives on how to provide early access to drugs while facilitating evidentiary certainty. Despite not being asked about clinical trials explicitly, many participants likened access to an approved drug with uncertain benefits to participation in a clinical trial. As one noted, “Even if [a drug] is approved, it’s still a trial,” while others described it as “more like testing.” Some participants distinguished between access to new drugs and FDA approval, questioning whether access could be facilitated without full approval, although no specific mechanisms were mentioned. Clinical trials were often seen as a way to address both unmet need and evidence generation, but participants stressed that more trials and broader access were required. As 1 participant summarized, “There should be more clinical trials available to different demographics of people…more test subjects…more accuracy.” Supportive quotes for theme 6 are provided in Table 4.

## Discussion

In this qualitative study, 30 participants with breast cancer in the US described how they weighed faster FDA drug approval against evidentiary uncertainty and when a trade-off was most acceptable. For many participants, additional uncertainty about clinical benefit was an acceptable trade-off for faster approval when facing end-of-life decisions, when no alternative treatment options existed, or when the anticipated benefit of a new drug was transformative. These values are consistent with the intent of the FDA’s Accelerated Approval pathway, which aims to facilitate faster access to new drugs that address unmet medical need for serious conditions, particularly for which no therapies exist or a new therapy offers meaningful advantages, such as novel or improved clinical outcomes, fewer adverse effects, or better tolerability.^3^

However, at earlier stages of disease, when treatment options are available, or when the anticipated benefit of a new drug may only be marginal, participants suggested that greater certainty should often take priority over speed. These findings align with evidence that individuals in the US with experience of cancer may be willing to wait substantial periods for clearer data on survival benefit.^7^ When weighing this trade-off, participants reaffirmed that overall survival and quality of life were outcomes that mattered most.^4^ For some, improvement in survival was the only outcome worth risking additional toxic effects for, while others viewed improved survival as meaningful only if functional status was also maintained. Yet, most breast cancer drugs granted accelerated approval over the past 2 decades targeted indications with existing treatment options, and most showed little or no improvement in overall survival or quality of life.^12^ Similar uncertainty has been observed across other tumor types.^13,14^

Participants also emphasized the added burden of drugs approved with uncertain benefit, particularly when layered onto already intensive treatment regimens. This concern aligns with evidence that patients with advanced cancer spend considerable time in health care contact,^17^ with contact days increasing over time as treatment options expand, particularly in breast cancer.^18^ Small survival gains may be offset by time toxicity and reduced quality of life from additional treatment.^19,20^ When meaningful benefit is uncertain or alternative treatments exist, even small additional risks mattered to participants. Yet, cancer drugs are often approved despite offering little or no clinical benefit^13,14,21^ while posing added risks of adverse events,^22^ financial toxicity,^23,24^ and the time burden of additional cancer care.^25^

Participants readily understood the trade-off between faster approval and evidentiary certainty, with expectations that largely matched their clinical circumstances. Such an understanding contrasts with earlier evidence that patients with metastatic cancer often believe that treatment intent could be curative,^26^ which may reflect improved patient education and health literacy,^27^ improved access to information in recent years,^28^ or comparatively longer survival of patients with breast cancer that allows patients more time to engage with their prognosis.^29,30^ These findings support prior calls for clearer information on the uncertainties surrounding newly approved cancer drugs.^31,32,33^ Given that many key uncertainties are not reported to clinicians in FDA-regulated information, clinical trial reports, and treatment guidelines^34,35^ and that physicians themselves often have a limited understanding of FDA approval standards, greater transparency could also benefit clinical decision-making.^36,37^

Several participants suggested that when uncertainty is high, access through clinical trials, rather than regulatory approval, may address unmet needs while also generating robust evidence, a dual benefit that has been discussed previously.^38^ Despite favorable views on clinical trials as a means of early access, participants described inequities that limited their personal participation and reflected on systemic barriers for rural populations, heavily pretreated patients, and minority groups. These experiences align with findings of previous research^39,40,41^ and represent a missed opportunity to simultaneously address unmet need and strengthen the evidence base before approval.

Several structural features of the Accelerated Approval pathway may contribute to the mismatch between patient preferences and current approval practices. Patients found uncertainty most acceptable when no alternative treatments existed and least acceptable when drugs with similar safety and efficacy profiles were already available. Yet, because drugs are considered available therapy only if they have received regular (not accelerated) approval, the FDA may grant accelerated approval to multiple drugs in the same indication,^3^ allowing fast followers to enter the market with limited data. This situation is particularly concerning given that more than one-third of oncology indications receive accelerated approval, with many retaining this status for several years.^42^ The FDA should consider reclassifying existing accelerated approvals as available therapy, thereby limiting pathway eligibility to drugs that genuinely address unmet needs.^43^ Patients also found uncertainty most acceptable if drugs were likely to offer transformative improvements in survival or quality of life. Yet recent evidence has shown that survival or quality-of-life improvements are uncommon, rarely occur together, and are modest when present.^21,44^ Breakthrough designation, which is associated with greater survival benefit^45^ and higher rates of conversion to full approval,^46^ may offer one way to operationalize transformative benefit as a pathway eligibility criterion for accelerated approval. Where demonstrating survival benefit before approval is not feasible, clinical benefit scales may help identify drugs most likely to improve patient outcomes, with recent evidence suggesting that these tools may distinguish drugs more likely to be verified as clinically beneficial in confirmatory trials and convert to full approval sooner.^46,47^

Additional research is needed to assess how this trade-off is viewed by individuals with different tumor types (from more indolent to more aggressive), stages (both curative and noncurative), and therapeutic availability (particularly in cancers with limited or no treatment options). Understanding how the clinical circumstances common to rare disease, including high unmet need, limited treatment alternatives, and greater uncertainty from small trial populations and trial design, shape these preferences is also important.^48^ Beyond oncology, it will be important to assess whether this trade-off is viewed differently in other chronic conditions that carry their own distinct clinical and evidentiary challenges.

### Limitations

This study had several limitations. It recruited patients with breast cancer with diverse sociodemographic and clinical characteristics but was not intended to be representative of all patients with breast cancer, and its qualitative design limits generalizability. As with any opt-in interview study, some self-selection bias is possible, although recruitment materials did not reference the FDA or drug approval to minimize this (eFigure in Supplement 1). Although 8% to 25% of patients with cancer in the US participate in support groups,^49,50,51^ recruiting through these groups could have introduced bias, identifying patients with greater health literacy and knowledge about their prognosis than the broader breast cancer population. To mitigate this limitation, support-oriented (rather than advocacy-based) groups were targeted, and participants disclosed relevant training or affiliations. Only 5 reported such involvement, all limited in scope.

Focusing on breast cancer also limits generalizability to other tumor types. Patients with breast cancer generally have broader treatment availability^52^ and better outcomes of metastatic disease^53^ compared with those with many other cancers, and nearly 90% of oncology accelerated approvals are in noncurative settings^54^ compared with 43% among patients with metastatic cancer in this study. Furthermore, only 2% of breast cancer approvals receive orphan designations, characterized by disease rarity and often higher uncertainty, vs more than 60% of all accelerated approvals.^12,46,55^ This proportion is particularly relevant given that accelerated approvals represent a greater share of approvals for other tumor types, eg, approximately 1 in 3 compared with 1 in 5 in breast cancer.^12,56^

## Conclusions

This qualitative study found that favoring faster approval over evidentiary certainty may be most acceptable among patients with breast cancer when no treatment alternatives exist or when anticipated benefits are likely to be transformative. When clinical benefit was uncertain, study participants emphasized survival and quality of life as priority outcomes and weighed the added risks of adverse effects and treatment burden. These findings reveal a mismatch between patient values and the common features of many FDA-approved cancer drugs. To better align regulatory approvals with patient values, use of the Accelerated Approval pathway may be most appropriate for drugs that address genuine treatment gaps or are likely to offer meaningful improvements in clinical outcomes over existing alternatives.

## Notes

Supplement 1. **eFigure.** Study Advertisement **eTable 1.** Full Interview Guide **eTable 2.** Individual Participant Characteristics **eTable 3.** Additional Illustrative Supportive Quotations for Subthemes

Supplement 2.
Data Sharing Statement

## References

[1] What we do. US Food and Drug Administration. 2024. Accessed November 10, 2025. https://www.fda.gov/about-fda/what-we-do

[2] Chen EY, Joshi SK, Tran A, Prasad V. Estimation of study time reduction using surrogate end points rather than overall survival in oncology clinical trials. JAMA Intern Med. 2019;179(5):642-647. doi:10.1001/jamainternmed.2018.8351 30933235 PMC6503556

[3] Expedited programs for serious conditions: drugs and biologics. US Food and Drug Administration. 2014. Accessed November 10, 2025. https://www.fda.gov/regulatory-information/search-fda-guidance-documents/expedited-programs-serious-conditions-drugs-and-biologics

[4] Booth CM, Sengar M, Goodman A, . Common sense oncology: outcomes that matter. Lancet Oncol. 2023;24(8):833-835. doi:10.1016/S1470-2045(23)00319-4 37467768

[5] Fashoyin-Aje LA, Mehta GU, Beaver JA, Pazdur R. The on- and off-ramps of oncology accelerated approval. N Engl J Med. 2022;387(16):1439-1442. doi:10.1056/NEJMp2208954 36129992

[6] Demonstrating substantial evidence of effectiveness for human drug and biological products. US Food and Drug Administration. 2019. Accessed November 10, 2025. https://www.fda.gov/media/133660/download

[7] Forrest R, Lagarde M, Aggarwal A, Naci H. Preferences for speed of access versus certainty of the survival benefit of new cancer drugs: a discrete choice experiment. Lancet Oncol. 2024;25(12):1635-1643. doi:10.1016/S1470-2045(24)00596-5 39571597

[8] Cancer stat facts: female breast cancer. National Cancer Institute, Surveillance, Epidemiology, and End Results Program. 2025. Accessed November 10, 2025. https://seer.cancer.gov/statfacts/html/breast.html

[9] Breast cancer statistics. American Cancer Society. 2025. Accessed November 10, 2025. https://cancerstatisticscenter.cancer.org/types/breast

[10] Slamon DJ, Leyland-Jones B, Shak S, . Use of chemotherapy plus a monoclonal antibody against HER2 for metastatic breast cancer that overexpresses HER2. N Engl J Med. 2001;344(11):783-792. doi:10.1056/NEJM200103153441101 11248153

[11] Swain SM, Shastry M, Hamilton E. Targeting HER2-positive breast cancer: advances and future directions. Nat Rev Drug Discov. 2023;22(2):101-126. doi:10.1038/s41573-022-00579-0 36344672 PMC9640784

[12] Michaeli JC, Michaeli T, Trapani D, . Breast cancer drugs: FDA approval, development time, efficacy, clinical benefits, innovation, trials, endpoints, quality of life, value, and price. Breast Cancer. 2024;31(6):1144-1155. doi:10.1007/s12282-024-01634-x 39320645 PMC11489271

[13] Gyawali B, Hey SP, Kesselheim AS. Assessment of the clinical benefit of cancer drugs receiving accelerated approval. JAMA Intern Med. 2019;179(7):906-913. doi:10.1001/jamainternmed.2019.0462 31135808 PMC6547118

[14] Liu ITT, Kesselheim AS, Cliff ERS. Clinical benefit and regulatory outcomes of cancer drugs receiving accelerated approval. JAMA. 2024;331(17):1471-1479. doi:10.1001/jama.2024.2396 38583175 PMC11000139

[15] Tong A, Sainsbury P, Craig J. Consolidated Criteria for Reporting Qualitative Research (COREQ): a 32-item checklist for interviews and focus groups. Int J Qual Health Care. 2007;19(6):349-357. doi:10.1093/intqhc/mzm042 17872937

[16] Braun V, Clarke V. Using thematic analysis in psychology. Qual Res Psychol. 2006;3(2):77-101. doi:10.1191/1478088706qp063oa

[17] Gupta A, Johnson WV, Henderson NL, . Patient, caregiver, and clinician perspectives on the time burdens of cancer care. JAMA Netw Open. 2024;7(11):e2447649. doi:10.1001/jamanetworkopen.2024.47649 39602118 PMC12040224

[18] Gupta A, Jazowski SA, Vaidya AU, Dusetzina SB, Ganguli I. Health care contact days in older adults with metastatic cancer. JAMA Netw Open. 2025;8(12):e2547924. doi:10.1001/jamanetworkopen.2025.47924 41364432 PMC12690425

[19] Gupta A, O’Callaghan CJ, Zhu L, . Evaluating the time toxicity of cancer treatment in the CCTG CO.17 trial. JCO Oncol Pract. 2023;19(6):e859-e866. doi:10.1200/OP.22.00737 36881786 PMC10337749

[20] Gupta A, O’Callaghan CJ, Zhu L, . The association of health-care contact days with physical function and survival in CCTG/AGITG CO.17. J Natl Cancer Inst. 2024;116(8):1313-1318. doi:10.1093/jnci/djae077 38656931

[21] Michaeli DT, Michaeli T. Overall survival, progression-free survival, and tumor response benefit supporting initial US Food and Drug Administration approval and indication extension of new cancer drugs, 2003-2021. J Clin Oncol. 2022;40(35):4095-4106. doi:10.1200/JCO.22.00535 35921606

[22] Richardson NC, Kasamon Y, Pazdur R, Gormley N. The saga of PI3K inhibitors in haematological malignancies: survival is the ultimate safety endpoint. Lancet Oncol. 2022;23(5):563-566. doi:10.1016/S1470-2045(22)00200-5 35429996

[23] Khan HM, Ramsey S, Shankaran V. Financial toxicity in cancer care: implications for clinical care and potential practice solutions. J Clin Oncol. 2023;41(16):3051-3058. doi:10.1200/JCO.22.01799 37071839

[24] Ehsan AN, Wu CA, Minasian A, . Financial toxicity among patients with breast cancer worldwide: a systematic review and meta-analysis. JAMA Netw Open. 2023;6(2):e2255388. doi:10.1001/jamanetworkopen.2022.55388 36753274 PMC9909501

[25] Gupta A, Eisenhauer EA, Booth CM. The time toxicity of cancer treatment. J Clin Oncol. 2022;40(15):1611-1615. doi:10.1200/JCO.21.02810 35235366

[26] Weeks JC, Catalano PJ, Cronin A, . Patients’ expectations about effects of chemotherapy for advanced cancer. N Engl J Med. 2012;367(17):1616-1625. doi:10.1056/NEJMoa1204410 23094723 PMC3613151

[27] Holden CE, Wheelwright S, Harle A, Wagland R. The role of health literacy in cancer care: a mixed studies systematic review. PLoS One. 2021;16(11):e0259815. doi:10.1371/journal.pone.0259815 34767562 PMC8589210

[28] Hopkins AM, Logan JM, Kichenadasse G, Sorich MJ. Artificial intelligence chatbots will revolutionize how cancer patients access information: ChatGPT represents a paradigm-shift. J Natl Cancer Inst Cancer Spectr. 2023;7(2):pkad010. doi:10.1093/jncics/pkad010 36808255 PMC10013638

[29] Cancer facts & figures. American Cancer Society. 2025. Accessed November 10, 2025. https://www.cancer.org/research/cancer-facts-statistics/all-cancer-facts-figures/2025-cancer-facts-figures.html

[30] Berger O, Grønberg BH, Loge JH, Kaasa S, Sand K. Cancer patients’ knowledge about their disease and treatment before, during and after treatment: a prospective, longitudinal study. BMC Cancer. 2018;18(1):381. doi:10.1186/s12885-018-4164-5 29614997 PMC5883273

[31] Herder M. Reviving the FDA’s authority to publicly explain why new drug applications are approved or rejected. JAMA Intern Med. 2018;178(8):1013-1014. doi:10.1001/jamainternmed.2018.3137 29971404

[32] Davis C, Wagner AK, Mintzes B, Scowcroft H, Woloshin S, Naci H. Patients deserve better information on new drugs. BMJ. 2024;387:e081720. doi:10.1136/bmj-2024-081720 39471990

[33] Coles CE, Earl H, Anderson BO, ; Lancet Breast Cancer Commission. The Lancet Breast Cancer Commission. Lancet. 2024;403(10439):1895-1950. doi:10.1016/S0140-6736(24)00747-5 38636533

[34] Cherla A, Woloshin S, Wagner AK, . New cancer drug approvals: less than half of important clinical trial uncertainties reported by the FDA to clinicians, 2019-22. Health Aff (Millwood). 2025;44(7):830-838. doi:10.1377/hlthaff.2024.01134 40623257

[35] Cherla A, Wagner AK, Wouters OJ, . Reporting of clinical trial uncertainties with new cancer drugs in journal publications and clinical guidelines. JAMA. 2025;334(16):1480-1482. doi:10.1001/jama.2025.13917 40900610 PMC12409641

[36] Dhruva SS, Kesselheim AS, Woloshin S, . Physicians’ perspectives on FDA regulation of drugs and medical devices: a national survey. Health Aff (Millwood). 2024;43(1):27-35. doi:10.1377/hlthaff.2023.00466 38190596

[37] Kesselheim AS, Woloshin S, Eddings W, Franklin JM, Ross KM, Schwartz LM. Physicians’ knowledge about FDA approval standards and perceptions of the “breakthrough therapy” designation. JAMA. 2016;315(14):1516-1518. doi:10.1001/jama.2015.16984 27115269

[38] Lynch HF, Bateman-House A. Facilitating both evidence and access: improving FDA’s accelerated approval and expanded access pathways. J Law Med Ethics. 2020;48(2):365-372. doi:10.1177/1073110520935352 32631197

[39] Unger JM, McAneny BL, Osarogiagbon RU. Cancer in rural America: improving access to clinical trials and quality of oncologic care. CA Cancer J Clin. 2025;75(4):341-361. doi:10.3322/caac.70006 40146038 PMC12223359

[40] Swenson WT, Swenson A, Westergard E, Schroeder Z. Geographic distribution of clinical trials for advanced-stage cancer. JAMA Oncol. 2024;10(8):1132-1133. doi:10.1001/jamaoncol.2024.1690 38829300 PMC11148782

[41] Kirkwood MK, Schenkel C, Hinshaw DC, . State of geographic access to cancer treatment trials in the United States: are studies located where patients live? JCO Oncol Pract. 2025;21(3):427-437. doi:10.1200/OP.24.00261 39356976 PMC11925346

[42] Beaver JA, Pazdur R. “Dangling” accelerated approvals in oncology. N Engl J Med. 2021;384(18):e68. doi:10.1056/NEJMp2104846 33882220

[43] Patel SR, Ramachandran M, Ai A, Chen CT. Redefining available therapy in oncology accelerated approval decisions. J Clin Oncol. 2025;43(6):629-632. doi:10.1200/JCO.24.00892 39454117

[44] Sherry AD, Miller AM, Parlapalli JP, . Overall survival and quality-of-life superiority in modern phase 3 oncology trials: a meta-epidemiological analysis. JAMA Oncol. 2025;11(7):718-724. doi:10.1001/jamaoncol.2025.1002 40451185 PMC12127959

[45] Michaeli DT, Michaeli T. Breakthrough therapy cancer drugs and indications with FDA approval: development time, innovation, trials, clinical benefit, epidemiology, and price. J Natl Compr Canc Netw. 2024;22(4):e237110. doi:10.6004/jnccn.2023.7110 38648855

[46] Tibau A, Hwang TJ, Romano A, . Factors in time to full approval or withdrawal for anticancer medicines granted accelerated approval by the FDA. JAMA Netw Open. 2025;8(3):e252026. doi:10.1001/jamanetworkopen.2025.2026 40136298 PMC11947834

[47] Cherny NI, Oosting SF, Dafni U, . ESMO-Magnitude of Clinical Benefit Scale version 2.0 (ESMO-MCBS v2.0). Ann Oncol. 2025;36(8):866-908. doi:10.1016/j.annonc.2025.04.006 40409995

[48] Michaeli T, Jürges H, Michaeli DT. FDA approval, clinical trial evidence, efficacy, epidemiology, and price for non-orphan and ultra-rare, rare, and common orphan cancer drug indications: cross sectional analysis. BMJ. 2023;381:e073242. doi:10.1136/bmj-2022-073242 37160306 PMC10167557

[49] Owen JE, Goldstein MS, Lee JH, Breen N, Rowland JH. Use of health-related and cancer-specific support groups among adult cancer survivors. Cancer. 2007;109(12):2580-2589. doi:10.1002/cncr.22719 17503435

[50] Sautier L, Mehnert A, Höcker A, Schilling G. Participation in patient support groups among cancer survivors: do psychosocial and medical factors have an impact? Eur J Cancer Care (Engl). 2014;23(1):140-148. doi:10.1111/ecc.12122 24106803

[51] Sherman AC, Pennington J, Simonton S, Latif U, Arent L, Farley H. Determinants of participation in cancer support groups: the role of health beliefs. Int J Behav Med. 2008;15(2):92-100. doi:10.1080/10705500801929601 18569127

[52] Etan T, Amir E, Tibau A, . National comprehensive cancer network recommendations for drugs without US food and drug administration approval in metastatic breast cancer: a cross-sectional study. Cancer Treat Rev. 2020;91:102113. doi:10.1016/j.ctrv.2020.102113 33128993

[53] Hortobagyi GN, Stemmer SM, Burris HA, . Overall survival with ribociclib plus letrozole in advanced breast cancer. N Engl J Med. 2022;386(10):942-950. doi:10.1056/NEJMoa2114663 35263519

[54] Tibau A, Romano A, Liu ITT, Han J, Cliff ERS, Kesselheim AS. Assessing outcomes emerging after conversion to regular approval for cancer drug indications granted accelerated approval, 1992-2021. J Natl Cancer Inst. 2025;117(10):2103-2111. doi:10.1093/jnci/djaf195 40690366

[55] Tibau A, Cliff ERS, Romano A, Borrell M, Molto C, Kesselheim AS. Predictors of withdrawal of anticancer drug indications granted accelerated approval: a retrospective cohort study. EClinicalMedicine. 2025;84:103088. doi:10.1016/j.eclinm.2025.103088 40687736 PMC12273735

[56] Scott EC, Baines AC, Gong Y, . Trends in the approval of cancer therapies by the FDA in the twenty-first century. Nat Rev Drug Discov. 2023;22(8):625-640. doi:10.1038/s41573-023-00723-4 37344568

