# Supplementary material for: Patient Perspectives on Approval Speed vs Evidentiary Certainty in US Cancer Drug Approvals
Source: JAMA Netw Open. 2026 Jun 9;9(6):e2617450. doi: 10.1001/jamanetworkopen.2026.17450 (PMC13250706; doi:10.1001/jamanetworkopen.2026.17450)
Supplement: Supplement 1. — eFigure. Study Advertisement eTable 1. Full Interview Guide eTable 2. Individual Participant Characteristics eTable 3. Additional Illustrative Supportive Quotations for Subthemes [file jamanetwopen-e2617450-s001.pdf]

Supplemental Online Content

Forrest R, Aggarwal A, Tregear M, et al. Patient perspectives on approval speed vs evidentiary certainty in US cancer drug approvals. *JAMA Netw Open*. 2026;9(6):e2617450. doi:10.1001/jamanetworkopen.2026.2617450

eFigure. Study Advertisement
eTable 1. Full Interview Guide
eTable 2. Individual Participant Characteristics
eTable 3. Additional Illustrative Supportive Quotations for Subthemes

This supplemental material has been provided by the authors to give readers additional information about their work.

© 2026 Forrest R et al. *JAMA Network Open*.

eFigure. Study Advertisement

Opportunity to take part in paid research interview

Supported by the LSE Knowledge Exchange and Impact fund

Study title
Investigating the views of individuals diagnosed with breast cancer on cancer drug approval in the United States.

Overview
This is an opportunity to take part in an interview study. The study aims to better understand the views of individuals diagnosed with breast cancer on drug regulatory approval. The study is led by researchers at The London School of Economics (UK) and Harvard Medical School (US).

Who are we looking for?
We are looking for individuals (aged 18 and over) that have been diagnosed with breast cancer.

What would be required?
To participate in the study, you would first fill out a short registration form providing some information about yourself and your cancer diagnosis (linked below). If you are selected to take part, we will contact you to schedule a 1-hour interview (online). To take part, you would need to be able to use Zoom/Teams to a basic level.

When would the interviews take place?
The interviews would take place between January and March 2025.

What information would be collected?
The interview would focus on understanding your views on different aspects of cancer drug approval in the United States. No existing knowledge is required. Any information or views collected in the study will be kept anonymous.

Would you be paid?
You would be provided with a $50 gift-card for participating in the interview.

What will information from the interviews be used for?
We plan to share these views in multiple forms including a research publication, conference presentation and a short summary report. We will share any study publications with participants.

To register your interest, please click the link below to a short registration form. The form should take approximately 5 minutes to complete.

LINK TO REGISTRATION FORM

If you have any questions, please contact:

Robin Forrest (researcher) via email: [email]

© 2026 Forrest R et al. *JAMA Network Open*.

eTable 1. Full Interview Guide

Topic 1: Current patient understanding of the drug approval process and expectations of FDA-approved drugs

Before a new drug can be prescribed to patients in the United States, what processes do you think occur?

- Who is responsible for assessing and approving new drugs before they can be prescribed? What are they looking for?
- What does approved mean to you?
- Is the approval process for all new cancer drugs the same? Are there different types of approvals and do you know what they mean?

If you were deciding about whether to take a cancer drug that had just been approved by the FDA, what would you want to know?

- Do you think about whether the drug is safe? What does safe mean to you?
- Do you think about whether the drug works? In what way? What does work mean to you?
- Do you think about how the drug might make you feel?
- Which of these are most important to you and why?
- What do you expect of drugs that receive FDA approval?

Clarification points:
For the next questions, I would like you to think about recently-approved cancer drugs, by this, I mean prescription drugs that have been approved within the two years.

Reiterate:
For these next questions I will focus on the Food and Drug Administration (FDA). This is the Federal agency responsible for approving new drugs in the US. From now on, when I ask about "approvals" or "approved", I mean approved by the FDA in the United States.

Topic 2: Patient preferences for certainty of clinical (survival) benefit versus speed of access

Once a drug is approved by the FDA, what sort of gaps in knowledge or uncertainties can still exist about how safe the drug is or how well it works?

- Once a drug is approved by the FDA, do you think physicians are certain about how well new drugs work?
- Even after approval by the FDA, what may physicians still be uncertain about?
- Do you think all cancer drugs approved by the FDA help patients feel better or live longer?

When do you think it is reasonable for the FDA to approve new cancer drugs without certainty that they will help patients to live longer, or feel better?

- How about if this meant the drug could be approved early, there were no available treatments, or if patients are really sick?
- When else might it be reasonable for the FDA to approve new cancer drugs without certainty they will help patients to live longer, or feel better?
- Is early access and faster drug approvals always better? Why?

© 2026 Forrest R et al. *JAMA Network Open*.

How would you feel about waiting for greater certainty that new cancer drugs work before they are approved by the FDA?

- Do you think everyone would feel the same as you?
- What factors would influence whether you would be willing to wait longer to know a drug works?
- How should the FDA decide whether to approve a new cancer drug or wait a bit longer for greater certainty?

Topic 3: FDA performance, balance and future outlook

Given everything we have talked about, do you think the FDA finds the right balance between approving drugs fast enough, and making sure they work for patients?

- Do you think the FDA does a good job? Why?
- Is there anything you would do differently if you were in charge of the FDA?
- What would your priorities be for approving new cancer drugs in the US?

Open question

Is there anything we haven't discussed today you would like to tell us about or ask?

© 2026 Forrest R et al. *JAMA Network Open*.

eTable 2. Individual Participant Characteristics

ID	Age	Race/ ethnicity	Education	US State	income	Health coverage (at diagnosis)	Year of diagnosis	Breast cancer stage (at interview)	Active treatment	Surgery	Radiotherapy	Chemotherapy	Hormone	Immunotherapy	Self-reported understanding	Training	Affiliations
1	40-49	Black or African American	College degree	*	Prefer not say	Medicaid, Medicare	*	Metastatic (IV)	✓	×	×	✓	✓	✓	3	×	×
2	40-49	Asian or Asian American	College degree	*	$20,000–39,999	Medicaid, Employer	*	NED	×	✓	×	✓	×	✓	1	×	×
3	40-49	Hispanic or Latino	Graduate degree	*	$80,000–99,999	Uninsured	*	Metastatic (IV)	✓	×	×	×	✓	✓	4	✓[1]	×
4	60-69	White or European American	Some college	*	≥$100,000	Employer	*	Early-stage (I/II)	×	✓	✓	✓	✓	✓	3	×	✓[2]
5	30-39	White or European American	Graduate degree	*	$80,000–99,999	Uninsured	*	Locally advanced (III)	✓	×	×	✓	×	×	2	×	×
6	30-39	White or European American	Graduate degree	*	≥$100,000	Employer	*	Early-stage (I/II)	✓	×	×	✓	×	×	4	×	×
7	30-39	Asian or Asian American	Graduate degree	*	Prefer not say	Employer	*	Early-stage (I/II)	✓	✓	×	✓	×	×	2	×	×
8	20-29	Asian or Asian American	Graduate degree	*	Prefer not say	Employer	*	Locally advanced (III)	×	✓	✓	✓	✓	✓	3	×	×
9	40-49	Black or African American	Graduate degree	*	$20,000–39,999	Employer	*	Early-stage (I/II)	✓	✓	✓	✓	✓	×	2	×	×
10	30-39	White or European American	College degree	*	≥$100,000	Military or VA	*	Metastatic (IV)	✓	✓	✓	✓	✓	✓	3	×	×
11	50-59	White or European American	College degree	*	<$20,000	Individual	*	Metastatic (IV)	✓	✓	×	✓	×	✓	1	×	×
12	50-59	White or European American	Some college	*	$60,000–79,999	Employer	*	Metastatic (IV)	✓	✓	✓	✓	×	×	4	×	×
13	60-69	Asian or Asian American	College degree	*	$80,000–99,999	Employer	*	Early-stage (I/II)	×	✓	✓	×	✓	×	2	×	×
14	30-39	Black or African American	Graduate degree	*	$20,000–39,999	Medicaid	*	Locally advanced (III)	✓	✓	×	✓	✓	×	3	×	×
15	30-39	Hispanic or Latino	College degree	*	≥$100,000	Employer	*	Locally advanced (III)	×	✓	✓	✓	×	✓	3	×	×
16	50-59	White or European American	High school diploma	*	$80,000–99,999	Employer	*	NED	×	✓	✓	✓	✓	×	2	×	×
17	50-59	White or European American	High school diploma	*	Prefer not say	Individual	*	Metastatic (IV)	✓	✓	✓	✓	×	✓	1	×	×
18	20-29	White or European American	College degree	*	<$20,000	Medicaid	*	Locally advanced (III)	✓	×	×	✓	×	✓	2	×	×
19	30-39	Black or African American	Some college	*	$40,000–59,999	Medicare	*	Locally advanced (III)	✓	×	✓	✓	×	×	4	×	×
20	50-59	White or European American	Some college	*	$60,000–79,999	Employer	*	Metastatic (IV)	✓	✓	✓	✓	✓	✓	3	✓[1]	×
21	30-39	White or European American	Some college	*	$40,000–59,999	Medicaid	*	Locally advanced (III)	×	✓	✓	✓	✓	×	3	×	×
22	50-59	White or European American	High school diploma	*	$20,000–39,999	Employer	*	Metastatic (IV)	✓	✓	✓	✓	✓	✓	3	×	×
23	40-49	White or European American	Some college	*	≥$100,000	Employer	*	NED	×	✓	✓	✓	✓	✓	3	×	×
24	50-59	White or European American	College degree	*	$40,000–59,999	Employer	*	Metastatic (IV)	✓	×	✓	✓	×	✓	2	×	×
25	60-69	White or European American	Graduate degree	*	≥$100,000	Employer	*	Metastatic (IV)	✓	✓	✓	✓	✓	✓	4	×	✓[2]
26	70-79	White or European American	Some college	*	$80,000–99,999	Employer	*	Metastatic (IV)	✓	✓	✓	✓	✓	✓	3	✓[1]	×
27	60-69	White or European American	College degree	*	Prefer not say	Employer	*	Metastatic (IV)	✓	✓	✓	×	✓	✓	2	×	×
28	60-69	White or European American	Graduate degree	*	$80,000–99,999	Employer, Individual	*	Metastatic (IV)	✓	✓	✓	✓	✓	✓	3	×	×
29	60-69	White or European American	Some college	*	$60,000–79,999	Employer	*	NED	×	✓	✓	✓	✓	×	2	×	×
30	60-69	White or European American	College degree	*	≥$100,000	Employer	*	NED	✓	✓	✓	✓	✓	×	1	×	×

Notes: To maintain confidentiality, ages are presented in groups, and information on US State and year of diagnosis has been excluded, denoted by an asterisk (*).
Abbreviations: NED, No Evidence of Disease; VA, Veterans Affairs
Footnotes: [1] Self-reported receiving some training in drug approval either as a psychologist; during a clinical trial; or at a medical conference. [2] Self-reported an organisational affiliation as a trained pharmacist.

© 2026 Forrest R et al. *JAMA Network Open*.

eTable 3. Additional Illustrative Supportive Quotations for Subthemes

Underlined quotes are included in the manuscript.

Theme 1. Interpretations of FDA drug approval

Subtheme	Additional illustrative quotes (participant no.)
**1a) Awareness of approval pathways**	- "*I would think that there are different types of approvals because there are different types of drugs*" (P1)<br>- "*I cannot say with certainty that I know that there are different [approval pathways], but I imagine the approval process for chemotherapy is different than the approval process for immunotherapy*" (P6)<br>- "*I think there are different types, depending on the severity and the side effects*" (P7)<br>- "*I don't know about this one, honestly, but I would think there were different types of approval, but I don't know too much about it*" (P8)<br>- "*No, I don't know*" (P9)<br>- "*I have no idea, but I would actually guess that they're different*" (P10)<br>- "*I would guess that there are different but I don't know that for sure*" (P12)<br>- "*I would think that they would be different for different types of drugs.*" (P16)<br>- "*I would bet they would be different types, because some things were offered to me that was, like on a, oh, I can't remember what they called it was like a temporary approval to do a study, blah, blah, whatever. So I think that there are different levels of approval.*" (P17)<br>- "*I would assume there's different types of approval depending on, like, newer research and like, for example, I know with triple negative, there hasn't in the past been as many options for treatment. So maybe when those treatment options come out, they might be approved more quickly, so that they can be more readily available for people that haven't had those options in the past.*" (P18)<br>- "*I think that they're the same for cancer drugs. I think that they are, I'm not sure.*" (P24)<br>- "*I believe there's different types of approval, if I'm not mistaken, um, much like we saw with the COVID vaccines and how they did, like a rush type of approval.*" (P25)<br>- "*Okay, I know there's, you know, just the regular approval process, there's the Fast Track approval process, which means... having such good results that they submit to a request to get it fast tracked so it can be fully approved for patients.*" (P26)<br>- "*I would think it would be maybe different for pediatric drugs. You know, that's my thought, but I don't know*" (P29)<br>- "*I don't know. I really don't. I don't know. I would think you'd have to go through the same type of approval, but I don't know.*" (P30)
**1b) Meaning of FDA approval**	- "*I know that there's no such thing as a drug that is harmless or that has no fatality, right? But it's like, I would just assume that the FDA just makes sure that it's in state, it's safe in the sense that it would do more good than harm to more people.*" (P2)<br>- "*It means that there's enough science to back it up to where I know that it is safe and effective*" (P8)<br>- "*I would imagine that they are either meeting the standard of care or they're better than the standard of care. I can't imagine [the FDA] saying, Oh, this one works half as well. That's good. That's a good option. Let's put our money into that. So I would say it's either matching current standard of care drugs or it's better than what we have now.*" (P10)<br>- "*Approved means that they really researched the drug, and they've done the clinical trials, and they know how it's going to affect people, and they know, you know what side effects it's going to cause, and they know maybe the safety of the drug and some of the adverse reactions.*" (P16)<br>- "*I would hope that it's been properly tested and that it's, you know, fairly safe, and that There's a good chance it's going to help me, but I don't know that that is true.*" (P17)<br>- "*For it to be approved, it just means to me that it's safe and approved, and that studies have have proved that the risk outweighs the or the benefit that outweighs the risk.*" (P23)<br>- "*It means that it's gone through all of you know the checkpoints. I was on one new, extremely new drug, and unfortunately, it didn't work. So I guess I'm still a little bit leery once it's approved, until they've had a little bit more time with it in the actual market.*" (P25)<br>- "I used to have an expectation that [FDA approval] means that it's probably something better than an older medication that was out there... But I have been finding that that is not as true as I've gotten further along and I've had more treatments" (P26)<br>- "*It means that my oncologist can prescribe it and I can take it.*" (P28)<br>- "*Approved means that, based on the trials and the outcomes that they see, that the drug overall, helps most people. I know it doesn't help all people, but it offers hope to, I would say 80% of the study.*" (P30)
**1c) Expectations of FDA approved drugs**	- "*you can't really have expectations because there's so much variety, there's like, not a surefire thing, right? The only surefire thing is that I will never get cured.*" (P3)

© 2026 Forrest R et al. *JAMA Network Open*.

	- "it's like, yes, I'm going through this, but it's also killing my cancer, you know. So it's just like, such a different mindset… I know that there's still going to be horrible [side] effects. I know that, like hell, it probably shortens life expectancy." (P5)<br>- "I expect, I do expect some side effects, but I expect to be able to safely take this medication with out worry of something greater than the typical side effects, like you may have body aches, or, I don't know, maybe skin discoloration, or, you know, but not something extreme" (P9)<br>- "I don't think that all [cancer drugs approved by the FDA] help [patients] feel better. It may help them, help them a little longer than maybe they were, you know, initially. Oh, but feel better. I don't I don't know." (P9)<br>- "I really, really hope so [that cancer drugs approved by the FDA]. I would say majority of them probably do [help patients to feel better or live longer], just because I feel like the testing for them is pretty significant." (P10)<br>- "To me, it would mean that it's either shrinking or eliminating the cancer or or at least maintaining no evidence of disease status" (P12)<br>- "I would, in my opinion, I would say they'd have to see a minimum of 50% efficacy in, in what, you know, what type of cancer they're trying, trying to eliminate, or beat or work for. So I, I would say 50%." (P12)<br>- "I still do not believe that every drug is for me. Okay, irrespectful the fact that they've been approved." (P14)<br>- "No, I don't [think that all FDA approved drugs help people to feel better or live longer]. I don't. I think that some of them, no, no, I think I don't. I don't believe that. I don't think so." (P17)<br>- "So just because they approved, it doesn't really mean that everyone's gonna like, get better and you're going to be fine." (P19)<br>- "Yeah, I would think if I'm taking it, it's supposed to work. I mean, they always tell you there's a chance it might not for whatever reason. But, um, yeah, I would expect if I'm taking it, that I it would work" (P20)<br>- "I mean, I think even if it's approved, it's going to come with side effects, but I would assume that the success rate is good if it is approved" (P21)<br>- "These drugs, if they are newly approved, I would expect lesser side effects than already on the market cancer drugs." (21)<br>- "So sometimes I feel like it's just, it depends on how your body responds. I mean, ultimately, it's that we're not going to live forever, and you can give all the medicines that you want, but if the body's not going to work, it's just not going to work, No matter what you throw at it." (P22)<br>- "Well, I would hope that they [the FDA] would be certain that everything worked appropriately, or they wouldn't release it." (P24)<br>- "Well, it doesn't mean that there is no risk… [I think] they make, a decision that the potential benefits outweigh the risks." (P28)<br>- "Number one, I assume it's not going to kill me outright, but I don't assume that there will not be side effects that I can't handle." (P29)<br>- "I would hope that it would be effective for what it's designed to do, um, and I would expect that the side effects would be what are disclosed" (P29)
**1d) Desire for greater transparency**	- "I don't even know what the processes are. Maybe that's what they need to do a better job of, of having their processes and stuff being more accessible to people, so that people are actually aware" (P2)<br>- "I think just if they can make the process a little bit easier for lay people to understand, sometimes it can sound very complicated, so I think like if there can be more outreach to explain things… just like a summary of what it does. I don't want a lot of details, but, you know, like a TLDR on like, this is how it was studied. This is how many people who studies these were the concerns, and this is why we made this determination" (P7)<br>- "…my oncologist is like, she brought out the actual study, and she explained the results of it. I'm not sure if all doctors do that, but I think like pulling out the studies, the original studies, and what the FDA originally approved it on, and explaining it to the patients and how it can benefit them. Can really help… a simple breakdown of the results of the study to where like, a like the average patient can understand I think that would be helpful as well" (P8)<br>- "if I had one suggestion for [the FDA] it would be to make, like a site for people that is not in medical jargon. I think a lot of people are absolutely petrified of clinical trials, and while they can be scary, they're just meant to be helpful in the end of the day… my suggestion would be connect more with the people that you're approving these things for. That's what everybody wants, right?… Make it easier for people to understand and make it less scary. I think you probably [would] have more participants, and then maybe it wouldn't take so long." (P10)<br>- "I think it's good whenever they put out, like articles or anything like that, that I can clearly read through and say, like, oh, here was the you know significance, and who it did it help? Who did it not help? Where? If it's like very laid out and clear and it's explaining both sides" (P18)<br>- "I think it would be nice if they could come right out and say, you know, in this trial it lasted this long or and maybe if there's more options, but I guess that's not really up to them, because if there's not enough research and not enough new drugs, they just have to give you what they have." (P20)<br>- "I don't know how transparent that [the FDA] are. I mean, maybe they should have a consumer friendly website, which maybe they do even have, like, I haven't even looked at that… that kind of breaks things down. Like, when I look at a lot of reports through Google, they're very medical based, and I wish there was a way to break it down more for the consumer." (P20)

© 2026 Forrest R et al. *JAMA Network Open*.

	- *“[I want] the Good, the Bad and the Ugly... I want it all. I don't want just the good... I want all the information that way I can determine what's best for me, but then sometimes I don't know what's best for me. The doctor knows what's best for me.”* (P22)<br>- *“I think clearly, access to information is, you know, there's not, obviously, there's not a lot of clarity in the process.”* (P28)

**Theme 2. Living with uncertainty and trust in physicians**	
**Subtheme**	**Additional illustrative quotes** (participant no.)
**2a) Familiarity with uncertainty**	- *“I think it's just really a crapshoot. I mean, it might work for me. It might not work for me. I mean, I want to believe in it. You know what I mean, because they tested it. But everyone's DNA is different. That's how I feel. Everyone reacts to things different.”* (P4)<br>- *“a lot of times with these cancer drugs, they can't guarantee... your body is different than mine, you know what I mean. So it's like, the side effects... it's not a one size fits all... there's no guarantees, and if there's going to be - hell, you could end up getting a secondary cancer from it!”* (P5)<br>- *“I was placed on Tamoxifen, and... it's been around for forever. We're confident that this will work and actually failed it within like, two or three months... we expected it to be a, you know, easy to take drug, and it wasn't. It didn't do anything for me at all. So it's interesting when that kind of stuff happens.”* (P10)<br>- *“my oncologist has said several times, I don't like that drug, and it's one of the ones that is used all, all of the time.... Within the last few years, I think we've also seen a really big shift in what is the best you know, even from some of the newer ones that when they first came out, this is going to be the best one. This is the standard of care. And two, three years into it, it's fallen back two to three...”* (P10)<br>- *“Every time I go in for an infusion, I'm wondering what's going to happen... there's always a hesitation. Approved, 2 years, 10 years, 5 years, 20 years, I think you're putting something in your body that's foreign... drugs don't work sometimes, and then they do other times...it's uncertain”* (P11)<br>- *“just as our faces are different, so [are] some of the drugs, these drugs might work differently in different individuals. So drugs that might work for Person A might not work for Person B”* (P14)<br>- *“certain drugs I think work better for some people, and other drugs might not work as good, you know, for another person”* (P16)<br>- *“you know, we're going to try this drug, and then if that doesn't work, we're going to, we're going to try the next one and the next one. So sometimes I feel like it's just, it depends on how your body responds.”* (P22)<br>- *“I think that the trials kind of shows a basic outline of how long it works. But everybody's different, and everybody responds to the drugs differently.”* (P24)<br>- *“And my doctor, they're like... well, well, let's try it, and if it doesn't work, we can, you know, we can switch you to something else. You know, I have heard that a lot through my diagnosis too which is yucky.”* (P29)
**2b) Trust in physicians**	- *“my oncologist has been working with cancer and cancer patients and research 20 plus years. And I think within that time frame, um, I'm not saying that all doctors have, but it gives you a little bit of comfort that maybe he knows what he's talking about.”* (P1)<br>- *“[my physician] understands, like, how the science works behind it, and she has read enough of the study and understands enough to where, for the clinical trial itself, the side effects aren't too bad, and that I could live with it. And so because of her confidence... and because of my confidence in her, I was able to confidently take the drug.”* (P2)<br>- *“the word that came to my head... was confidence. Like, if you're confident, then I will be confident, because that's your wheelhouse. So if you're, you know, feeling like this is the next best option for me personally, then you know, I'm good to go. So I think confidence is really what I'm I'm looking for.”* (P10)<br>- *“personally with my oncologist, I would feel very comfortable with whatever she recommended, because she is so knowledgeable and does her research herself, so I would trust her”* (P12)<br>- *“and the fact that it's been recommended by my doctor, I would think that they have done their due process also in trying to check the efficacy or the effectiveness of this drug. So I would rely on my doctors to guide me” (P13)*<br>- *“I would place my trust and my doctor to tell me the pros and cons about it”* (P13)<br>- *“I mean, I read studies and, you know, I talked to my oncologist, who is right on top of everything. He's like the smartest human I've ever met in my life.”* (P17)<br>- *“I think I trust when my oncologist tells me that something is going to work, that I have to give it a chance”* (P18)<br>- *“I would say that they are fairly certain, or they wouldn't prescribe it”* (P21)<br>- *“my oncologist is pretty frank with me in saying, you know, it is a new drug, and over time, you know, we'll have better, a better feel for it... she has been very clear that, just because it's approved, that's not the end all and the be all for everything.”* (P26)<br>- *“I think you know, they've all done their homework, and they know that it works... they know the effectiveness in terms of the survival rate, increasing your survival rate by you know, such and such a percentage... they certainly know all of that.”* (P27)

© 2026 Forrest R et al. *JAMA Network Open*.

Theme 3. Factors shaping attitudes toward waiting and uncertainty	
**Subtheme**	**Illustrative quote** (participant no.)
**3a) Role of existing treatment options**	- *"when it's the last resort medication… they would rather try something than not have a chance at it at all."* (P1)<br>- *"I think if there's a drug that already treats that symptom or that cancer that we know is safe and effective and there's no reason to speed up the approval process where you might potentially be missing serious side effects or you need a bigger sample size to have more certainty. There's no reason to speed up approval."* (P6)<br>- *"I know that there are experimental drugs out there and that they're just beginning to try and there are absolutely patients that are, you know, stage four, like I am, and not having good luck with other medications… they're willing, they're willing to try something that's new and not as well known or researched, just an ability to try to try to live longer"* (P12)<br>- *"I think there are patients that would be willing to try [drugs with uncertain clinical benefits] if they've been on other prescriptions or drugs that haven't worked, I think other some patients would be willing to try, even if it hasn't been approved. I know if I were in that situation and I had tried something and it hadn't worked, and I'd been told, Well, there's this new drug that, you know may but we're not sure I would be willing to try… as long as they know the safety of the of the drug and there are no other treatments, then I think it would be okay to for [the FDA] to approve it and say, you know, we're not sure the success rate this drug will have, but we know it's safe and It's not going to have caused too many adverse reactions, then I think it would be okay."* (P16)<br>- *"You know, I think that's kind of a double edged sword. If there are other drugs available for that particular type of cancer that work, then I think they, you know, [the FDA] needs to be certain. But if there aren't many drugs to treat that certain type of cancer, then as long as it's safe, I think they need to go ahead and get it approved and try to use it so that there aren't people out there with without treatment at all."* (P16)<br>- *"if you have people out there that nothing else has worked for them. You know, I think it would be good for them to have it, but you don't want people you know leaning toward that drug. If there are other drugs that they know they're certain about."* (P16)<br>- *"I guess [my willingness to wait for greater certainty] would depend on whether I was one of those people that was told you don't have any options."* (P17)<br>- *"I think that the patients should absolutely know, hey, we fast tracked this, because you have no other options. And I think if anybody has other options, then I think they should choose those other options. And I don't think that they should be available this particular what, what you're talking about right there, that shouldn't be available to just anyone. They should only be available to those folks that the oncologist has said you're out of options. You don't have other options."* (P17)<br>- *"I think if, if there is some research to show that it can be helpful, and if there are no other alternatives, I think something is better than nothing… I'd much rather deal with that uncertainty than have no additional treatment option."* (P18)<br>- *"I think it would really depend on where I am in this journey, like, if, for some reason, this drug failed me and the next one did, then I would be like, the first in line and just say, give it to me like what does it matter at this point."* (P20)<br>- *"I think people with certain cancers that don't have drugs available to them specifically would be willing to take the uncertainty of it."* (P21)<br>- *"I think if I had run out of options, then I would be okay with it"* (P24)<br>-
**3b) Individual choice and treatment urgency**	- "*It's definitely not the same for everyone. That's why I said where, where I'm at, I would be comfortable waiting, but other people may not be as comfortable waiting. It just depends on where you're at in your cancer journey."* (P21)<br>- "*I do think that anyone who is facing a really, I guess, facing death, gets to choose, to be able to choose whether they want to take something new and revolutionary that doesn't necessarily have as many studies as they want, or if they want to not take it because there are certain side effects… any patient I believe should have access and appropriate knowledge… as long as they know that there's not as many trials or research that backs that it's still their decision. I think they should be allowed to make it*" (P23)<br>- "*You don't want to wait too long because your time runs out, right? So I think that it would have to be put in perspective naturally, as far as how long the wait is*" (P25)<br>- "*I can't wait for certainty. I have to be able to take my chance. So I think anybody that that has a time frame of knowing how long they have to go, I think are are willing to take their chances of taking a drug that isn't entirely proven because of the fact that they're they're desperate to be able to prolong their time.*" (P2)<br>- "*honestly, like, I don't know how long I'd wait. I mean, if it was like, if it was a situation where, okay, I just found out, I have to get my treatment right away… I'm not going to sit around for six months and wait for more data to come out… You don't just sit around when you have cancer."* (P4)<br>- *"I think with cancer, you don't really have much time to wait, so I think it would depend on where it falls in my treatment progress… there's not really much time to wait and say, well, I'm going to, I'm going to see how that works out with everyone else first, and then I'll consider it."*(P18)<br>- "I do think that should be left up to the patient. And I think that if you have a good doctor who can talk to you and say, like, this is newly approved. Like, I think it should happen quickly, because some people just don't have the time." (P20)

© 2026 Forrest R et al. *JAMA Network Open*.

**3c) Transformative versus incremental anticipated benefit**	- *"If there was a stage four cancer, stage four breast cancer drug that showed really promising results in early clinical trials, I would want that to be approved as quickly as possible. Um, if there was a new drug that's going to compete with like, the four other potential chemo therapies for early stage breast cancer, I might say, hold off unless it was showing, like, very minimal side effects or or if it could be used in people who can't do the other types of chemotherapies, or something like that... I think if it was a drug that like had similar results and similar side effects to drugs that are already approved, there's no reason to speed it up."* (P6)<br>- "*I guess it just really depends on the kind of cancer and situation, but like, if there were a breast cancer, and it's something like Herceptin, which is very common, and there's a new competing drug which doesn't do that much more, then maybe wait longer, but if it dramatically improves outcomes or something, then, you know, approve it and then see if people are interested*." (P7)<br>- *"I mean, if this, this thing that they create, comes and it's just working on every single person, and every single person lived for 20 more years, and it's guaranteed, then sure, push it through. But realistically, realistically speaking, I just think that if something comes through quicker, there's just room for error"* (P10)<br>- *"I mean, I think sometimes, if it's really going to be a breakthrough, we should push harder to get it approved... if they gave it to me, [and]I'd be guaranteed, like, 10 more years. 20 more years"* (P20)<br>- "*I would just say if, if the efficacy that, if you're seeing unbelievable results like what I've read in some of the vaccine trials, then I think it's worth pushing it forward*" (P23)
**3d) Risks of drugs with uncertain benefits**	- *"personally, I would feel better with more certainty. I wouldn't feel comfortable with somebody saying this, oh, this is about 50% approved. It hasn't been approved yet, but it's about 50% there. No, that wouldn't work for me... especially with cancer, you've experienced enough harm... you don't want to take anything that is going to be a detriment to your quality of life, that's for sure" (P9)*<br>- "it *may treat my breast cancer, but it might give me diabetes, you know. So it's weighing those pros and cons."* (P12)<br>- "*I mean, just because you are someone young and healthy, you know, and you fit the profile to take this drug does not mean that it won't potentially cause any damage or side effects."* (P15)<br>- "*I actually had a pretty significant side effects, so permanent damage to my organs because of this drug... I was 32 years old and had never had any major medical issues, you know, and given so much information, and it's kind of conflicted, conflicting because I do believe this drug saved my life... I don't have stage four cancer, but I now have to live, you know, with medical care for the rest of my life. So in, in knowing that about me, I would definitely want to do more research, like, how, like, what are those? What they call, like, less than 1% possibility side effects... I would definitely, you know, maybe reconsider my take, you know, partaking in this medication, and go with something less riskier*." (P15)<br>- "*Just because a drug has been approved... the risk of taking it is still the same, you know... I believe you should still, you know, do your risk assessment, and also, if anything, make sure that your your doctor does a full workup on you to make sure you're not at risk for a possibility of encountering any of these permanent side effects."* (P15)<br>- *"why am I going to take just like a random draw of the luck, you know, like something that could potentially actually kill me rather than help or extend my life? I need to be kind of certain that it will help me to live longer or feel better before approving it... you don't want to cause more damage than what is already there*." (P15)<br>- "*Like, okay, okay, this is gonna this is gonna help my cancer, but in the meantime, we're gonna suppress the hell out of your bone marrow, and your counts are going to be low. It's like, Well, how long do I live that way? You know, or your kidneys are gonna, you know, you'll be in dialysis because, you know, after taking the drug for 10 years, your kidneys are gonna not work well. But hey, you live 10 more years, right?"* (P22)<br>"*I would probably look into more of those kind of things and focus on the safety of what we're approving and not the speed."* (P29)
**3e) Individual factors influencing willingness to wait**	- "*if I'm stage four or metastatic, it's like, whatever you give me, that's an option that has even the slightest chance of bettering my position, I would take it right. But if I'm stage one and there's like more options, or there's a chance for me to wait for something else better to come along, then that's going to impact my decision*" (P2)<br>- *"If you're asking, like an 80 year old person, like, I don't think I would put myself through, yeah, I definitely don't... whereas, like, at 38 you know, I want, I hopefully have only half my life done that, like, I'm willing to put myself through a lot, you know"* (P5)<br>- "*are we talking about a super rare cancer where, like, you know, I have another friend who's, like, one of two people in the world with, like, a specific type of leukemia. So it's like, okay, well, in his case, I don't know, maybe [it is okay to approve drugs without more certainty of clinical benefit], but like, if you're gonna say, like, in breast cancer, I would say, hell no, because, like, that's, that's so many individuals. It's, it's a cancer where there's already, like, a ton of very, you know, effective treatments."* (P5)<br>- "*if you are metastatic, you don't have, like, I'm sure I would ask questions, but like... you're more desperate for something to extend your life, right? So I think your cancer stage plays a huge role in honestly, your openness [to accepting uncertainty]."* (P5)<br>- "*I think if it's like stage four, like I mentioned earlier, or if it's like a really rare like orphan disease, like enough to where it's debilitating for the patient, and the quality of life is not so good. I would think that in those instances that would be okay [to approve it with higher uncertainty of clinical benefit]*" (P8)

© 2026 Forrest R et al. *JAMA Network Open*.

	- "*you always want to help the next group of people that are unfortunately going to deal with this, but I still feel like two years would be, you know, my comfort amount of time. If I was older, it would probably be much longer than that. But two years [wait] for me is, I think, where I would stay.*" (P10)<br>- "*I think people, maybe my age or my circumstance would feel comfortable with I don't know, two to five years maybe, but I definitely think I don't know if I was in my 70s and I was dealing with this, I would just live as long as I could, because I've already lived life, you know, like you've already gotten through a chunk, a huge chunk, of your life, and especially if there are, if the young population is on the rise, or whatever, I would be fine with waiting however long I would Need to. But I think selfishly, being in my 30s with two small kids, two years feels like it still feels like an eternity, but I could do that*" (P10)<br>- "*I guess for myself, because I've had no evidence of disease for almost four years, I personally would be more hesitant to try something, but I know that people with the more advanced, advanced stage or place in their treatment, they would absolutely be more willing to try.*" (P12)<br>- "*I would probably think about where I am at the stage of whether would I be willing to take some more other risk associated with a drug, or do I think it would be really helpful to me, considering, you know, my age. I'm already almost, you know, 70s in the next few years… I think, you know, is it really necessary for me, just like when I the same approach that I would use if I if a doctor offers a surgical procedure to me and it said, Do I really need to have further surgery, considering my my age and my other medical conditions? Do I really want to subject myself to go through all the other things associated with the drug, I would consider that.*" (P13)<br>- "*You know, if I'm a young person, you know, I'll be willing to wait, if I'm an older person, and I don't have any other problems that I'm dealing with right now is that, you know, give it to me, but it depends, you know, it's going to be, you know, a personal choice*" (P13)<br>- "*It's definitely not the same for everyone. That's why I said where, where I'm at, I would be comfortable waiting, but other people may not be as comfortable waiting. It just depends on where you're at in your cancer journey.*" (P21)<br>- "*You don't want to wait too long because your time runs out, right? So I think that it would have to be put in perspective naturally, as far as how long the wait is*" (P25)<br>- "*hey, I would think, and I hate to say this, but maybe at stage four, when you're kind of at the end of the line, they would be more accepting of a new drug that is has uncertainty around it.*" (P21)<br>- "*I believe people, when they're at a certain diagnosis stage or their prognosis is not as good, then their feelings are, give me this. Let me try it.*" (P29)

**Theme 4. Balancing faster approval with evidentiary certainty**	
**Subtheme**	**Illustrative quote**<br>(participant no.)
**4a) Faster approval not always better**	Q: Is early access to new cancer drugs and faster drug approval always better?<br>- "*No. It's not always better. I mean, it's good, probably in some circumstances, but on the whole, I would say it's not good to rush a drug approval.*" (P4)<br>- "*not necessarily, because there are so many other ifs, you know, out there that they probably need to consider.*" (P13)<br>- "*I think not. I mean, I think it's great that they get the drugs out there as soon as they can, you know, for patients dealing with cancer, because, you know, it's a life or death. But I don't think the quickest way is always the best way, because they do need to go through the safeguards and the clinical trials*" (P16)<br>- "*I don't think so, because, you know, certain times I think the longer you look at something, the more sure you can be that it is helpful and not harmful. But like we just said, if it's compared to having no options, then probably yes, I'd prefer to have earlier access, and it be not studied as much like when I first was being treated.*" (P18)<br>- "*I probably feel in the very middle of the two extremes where I think a lot of people would think, no, faster, earlier is more dangerous. And I'm sure a lot of people think, oh, faster and earlier is, you know, basically getting in early. So I think probably there's a 5050, split on that one.*" (P18)<br>- "*No, I'm not for fast, that's for sure.*" (P25)<br>- "*No, not always. In some cases, some people may want them, the desperate people, the ones with bad prognosis, yeah, they're going to want that. But then again, I mean, if their prognosis is bad and they take a drug that's bad for them, they're really in the same boat, aren't they? I mean, so desperate people really have feel like they have less, less options. That's it, is the truth of it*"
**4b) Speed-certainty balance not always met by FDA**	Q: Do you think the FDA finds the right balance between approving new cancer drugs fast enough and making sure they work for patients?<br>- "*Um, yes, they do. It's a it's some nasty work. But, yeah, I, personally, I think that they do, they do the best that they can.*" (P1)<br>- "*Um, I mean, in my situation, I would say yes, but I know there's other people who are waiting for drugs to be approved, and maybe they go elsewhere for them. I mean, some people go to Europe, some people go to Mexico. You know what I mean for drugs that are approved. So I think in some circumstances, it takes too long, where people have to go other places for treatment*" (P4)

Subtheme	Additional illustrative quotes (participant no.)
	- "I think that there's a lot of things that could be faster out there... I'm sure it's a bureaucracy, and I know they want to be safe, um, but I think there's probably a lot of ... not inefficiencies, but like, blocks in the process... knowing how the government works... there is definitely room for efficiencies" (P5)<br>- *"I actually think that the FDA takes too long. I think it could do it quicker. So in terms of balance, my concern is more that it might take too long for the data to prove drugs."* (P7)<br>- "*Yes, I think they are a good governmental agency enough to where there's they are backed by signs and evidence to find that balance.*" (P8)<br>- "*So I think they do [find the right balance]. I think they do, or they try hard to, let me say that, but for the most part, yeah, I think so.*" (P9)<br>- *"I don't know... I would hope so. I mean, my good side of me says I would hope so. I mean, I would hope that that's like the end result is to make it so that we're all doing better and but... I feel there's a little, you know, I kind of mentioned the little corruption word. I mean, I think there is a little bit to be said about business and pressures... there's a lot of capitalism going on"* (P11)<br>- *"not always, not always. I think there are times that they put a drug on the market too soon, without knowing enough information."* (P12)<br>- "*Yeah, I feel so I feel that the FDA are doing just enough, you know, to make sure that the public is safe in approving drugs that are meant to be approved into the market, per se, okay, like I said in times past, I know that there, there have been times that drugs that were not meant to be approved, we actually approved. And then, and then the and then the target market really affected. But I also believe the modalities have been put in place.*" (P14)<br>- "*Oh, that's a really tough question. I tend to think when I hear that other countries have using this drug, have used this drug, and how many people, you know, and how it's really helped, and they're really, you know, fighting cancer or helping something else a lot better there, but we don't have this here. It tends to lead me to believe that they don't have a good balance, that some things could be approved faster*" (P16)<br>- *"No, I don't think so. I don't think so, because I think that they're pushed. They're I think, I think that the drug companies have a lot of influence, and probably more influenced than they should have... I think they're all corrupt... I think they're all together. I think that the FDA and the drug companies are like hand in hand. And I think that a lot of drug companies get what they want because they pretty much run the FDA. I'm pretty sure so."* (P17)<br>- "*For me, I would say, is not really balanced. I feel like you're trying to make everything so fast, yeah, like, you know, approving drugs very fast and not trying to make sure that it's going to, like, work on patient perfectly, and, you know, for them to get better*" (P19)<br>- "*Ah, um, no, I think they can always do better*." (P23)<br>- "*No, I think there's definitely it. It needs to be looked at carefully, and where can changes be made. I know even like during COVID, things went a lot faster during COVID for the vaccines and and there was more collaboration the scientific community.*" (26)<br>- "*I guess I'd have to say no because of the speed or lack of speed, or just the length of time it seems to take them to approve things.*" (27)<br>- "*It, it seems to me that there are an awful lot of drugs, not cancer drugs, an awful lot of drugs that I see on in commercial, ads, on my Facebook feed and social media, and there seems to be a new one every day. And I don't know that that's a good thing.*" (29)<br>- "*I feel like the FDA is very conservative and making sure that the medications that they're making available to the population is safe.*" (P30)
**4c) Skepticism about waiting longer**	- *"[waiting longer for greater certainty that a cancer drug works before approval] is a fallacy. That's not true because you don't know... there is no certain... nothing is certain."* (P1)<br>- "*the only benefit [of waiting longer] would be that they do have more information on it and how effective it would be, but then you're, you know, what if it's not effective? What if I wait, it's not as effective as I thought, and I wasted all that time*"(P4)<br>- "*Most of the people that are in the infusion room with me like if they can't get a treatment because their blood cultures are not right or something, they're extremely disappointed to be able to not get treatment. They you just when you have this [cancer] in you, you, just want it eradicated. So I don't know that everybody would [be willing to wait]*" (P25)<br>- *"Well, you know, when you have these time bombs in your body that are trying to kill you, Time matters. So I'm not quite sure it's a good thing to for them to wait."* (P27)<br>- *"I guess I need to understand what the definition of certainty is. I you know, because, like we discussed earlier, nothing is 100% so you should never be waiting for 100% because we're just not there in terms of cancer treatment."* (P28)<br>- "*[How would I have felt] about waiting? I think that would depend on my prognosis. But for me now, with my looking back and knowledge, I don't know that I would have wanted to wait. You know, probably not, you're in a catch 22 really?*" (P29)

Theme 5. Meaningful treatment outcomes

Subtheme	Additional illustrative quotes (participant no.)

© 2026 Forrest R et al. *JAMA Network Open*.

**5a) Survival**	- "if it was [a drug] for a metastatic cancer, how long it expands life expectancy" (P6)<br>- "*one of the reasons why I wanted to take the drug that was recommended to me was because the life expectancy for people that didn't take the control drug was less than the people that did. So it was like a difference of like, maybe, like 18 months or so. But even then, for me, I think I could do a lot in 18 months, or it could be longer, because the study is still ongoing after FDA approval.*" (P8)<br>- "*I think I was kind of persuaded by the results, [my physician] showed me the actual graph of the results of life expectancy for patients with and without it. And at that time, the FDA just approved it... [my physician] was like, the risk versus benefit of you not taking it could be months in the long run. And so for that, and for me, it it meant a lot. Because, like wrestling with cancer, you kind of think about it every day. So, like that, time is very precious*" (P8)<br>- "*I couldn't care less what the side effects are. Because I think when you're in this stage, you just you want time... side effects you can deal with*" (P10)<br>- "*You know, am I going to get two months more of life out of this, or am I going to get, you know, three years of life out of this? So I would like to know, you know, how long they've actually been testing it, and what their outcomes were*" (P25)<br>- *"You do have to see if it's going to extend life, and that takes time. There's no way that you can step around that... I don't know how they decide that, but that is definitely got to be hit, because I feel like that's the first thing that matters, and then the quality of life while it's extending the time... I just kind of can't wrap my head around why you'd make a decision to skimp on that when you try to are planning to seeing whether or not you should approve something, because those are the things that really matter at the end of the day to a cancer patient. And so if the drugs not delivering on that, and you just kind of put it out there and say, we're going to give this to you because we're going to approve it, because we think it might work. I think that's a problem."* (P28)
**5b) Quality of life and functioning**	- "*what's the point of having a drug for your cancer if it is no longer growing. Um, it stopped. It's you're in remission. But yet, your quality of life sucks because, well, now you're in congestive heart failure, or your your livers aren't functioning because there is so much toxicity in your body from all of these types of things that you you've done in these treatments the nerve pain is so severe that you can't you can't even drive to do anything anymore because you can't feel your feet, you can't write.*" (P1)<br>- "*my understanding is there is no cure at this moment. And so my object, you know, my objective with certain medications is stability, is going to be, you know, quality of life and all of those things, apparent functioning.*" (P3)<br>- "*What are the potential negatives of taking it, risks of taking it? What are the side effects? How are they weighing like, quality of life versus extension of life*?" (P6)<br>- "*But I think for me, in terms of time, I'm I'm thinking like, I can do a lot with that time, but also looking kind of at the side effects as well. In the study, there weren't a lot of side effects that were debilitating. So I'm really looking at, also at quality of life while taking the drug.*" (P8)<br>- *"I would be okay with [some uncertainty of clinical benefit], as long as it doesn't affect like activities of daily living. Like, am I still able to walk? Am I still able to eat, like, dress myself able? Am I still able to like, move around independently. I think those are pretty important to me."*<br>- "*My priorities would be for quality of life primarily and quantity of life secondary. I believe that quality of life is better than quantity of life, and that's what I have chosen [in my treatment]... it would have to be quality before quantity. But they both kind of go hand in hand.*" (P17)<br>- *"[my treatment goals would be] probably minimal side effects. Um, because really, Interviewer, the the quality of life for cancer patient. You know, you can keep me alive for another six years, but if I can't get on the ground and play with my grandchildren, or I can't, you know, go for a walk with my husband, it's really not worth it, if that makes any sense"* (P25)

**Theme 6. Appropriate ways to facilitate faster access**	
**Subtheme**	**Additional illustrative quotes**<br>(participant no.)
**6a) Uncertain approvals likened to clinical trials**	- "*[taking a new cancer drug] is a trial. Because if it's not working like again, with myself... my doctor decides, okay, well... that's not doing your numbers are not whatever. It's still progressing. We're going to try something else. It is a trial to me. In my mind, it's a trial run... So even if it is approved, it's still a trial.*" (P1)<br>- "well, I mean, that that, to me, is a clinical trial... are they approved all the way? Did they go through all the stages? I don't know it's to me that's more like testing." (P4)<br>- *"Isn't that? Wouldn't that be part of a clinical trial?"* (P28)
**6b) Earlier approval versus other pathways**	- *"The FDA shouldn't approve something for for general public, but if something is available and a patient is willing to, and it's, you know, recommended by their doctor or whatever, then the patient should have access. I guess, having patient access versus having FDA approval do different things in my head, if that makes sense."* (P2)<br>- "*the patient should have the right to choose if they want to utilize something based on even if it does not have FDA approval. And the only way they could have access to that is by clinical trials, right? So then patient still has access to it because they're participating in a clinical trial, as opposed to being able to be just be freely prescribed that medication by their doctor because it was already FDA*

	approved. So I guess in that sense, the FDA should just continue doing what they're doing to make sure that for the greater good that medications are meeting the standards that they need to meet" (P2)<br>- *"[when thinking about the balance, the FDA needs to think about] who needs access to the drug to have positive outcomes versus who can wait, and if there's a way to do, I know there's not, like, I don't know what's called interim approval, or like, putting those people into a clinical trial where they can have access to that potential life saving drug, and making sure people aren't being prevented from having that access that truly need it in order, like, if it's their only hope for living, making sure they have access to it."* (P6)<br>- *"Maybe there's a way that these people can be given the drug without it being approved by the FDA. I'm not familiar with the inner workings of all of that, but I, my priority is that the people who need it should get it… I don't think it's like a blanket policy for all drugs. I think it really needs to be for, you know, life saving situations where there aren't other options. I don't think this needs to apply to like all cancer drugs, or all drugs in general. I think there it can be done in a more systematic way, with the understanding that we don't have anything else available. This is going to help save a life. We don't know everything about it, but we want people to have access to it."* (P7)<br>- "*what we really need to have an emphasis more on is access to clinical trials… it's so important for people to have access to clinical trials, because if they're, if everybody's waiting for the approval of the FDA, that's just so difficult."* (P2)
**6c) Inequities in clinical trial access**	- *"some [patients can access clinical trials], but not all of them. Think it really depends on where you where you're at… So I would say that if you unfortunately hear about a clinical trial that you were really interested in but your hospital or clinic doesn't offer it, you might not be able to get to it. You might have to commit to travel, and that's not feasible for everybody."* (P10)<br>- *"I think there should be more clinical trials available to different demographic of people, and that way there are more, you know, there are more test subjects there. There's more accuracy or more numbers of how this drug affects different people, and if it's doing its job."* (P15)<br>- [Q: how can the FDA decide whether to approve a drug now or wait longer for greater certainty. So how can the FDA find the right balance?] *"More clinical trials?… Yeah, so definitely more. If there are more studies, you know whether they be clinical or in a lab with like animals, you know, I think there needs to be more data. And I think maybe that is what potentially prolongs this waiting period, right? So if there are more, if there is more data available, so more tests being conducted, more clinical trials, therefore there are more there is more data, then the FDA should have the information, sufficient information enough to Clear this drug or not." (P15)*<br>- *"[on my priorities for cancer drug approvals] if there's any way to increase the amount of research or the amount of people that are, you know, invited to clinical trials, I think there's a lot of times where, like, for example, I signed up to be a part of clinical trials, and haven't been reached out to by anyone. So I don't know if there's a lack of clinical trials, or if there's a lack of clinical trials for my age group, or what it is, but maybe just increasing that research overall, and increasing those groups that we're giving the opportunity to to try out these drugs."* (P18)<br>- *"if it came to an experimental drug, you know, yes, then, and if I'm into that, hey, we don't have anything left for you. We would like to try this. I think I would be willing, because it's just going to help the next person. And I think that's why I have already participated in two clinical trials, because how else are we going to know without the information?"* (P22)<br>- *"When [my physician] at [X cancer facility] says, oh, you know, I've got this drug, new drug coming out. It's got good results. We're very excited, you know? Why can't I get that drug? Because it's not approved by FDA yet, you know? And I can't get in the trial because it's already started, or, I think in that case, I was already on a drug. So they think I would mess up the results, which I think is kind of odd, you know, like, why don't you have a group that's already on it, on something, and trial them against a population that's just getting it for the first time? So I think it's just that accessibility and the speed of getting in people's hands that need it are, you know, my concerns."* (P27)<br>- *"if it's in trials, it needs to be available to everyone. I don't think the trial should limit people. I think it should be available. I think the people themselves should be allowed to say yes or no, not to be told you don't qualify for this trial. I think if it's if it's if it's gone through the original studies and it's out there in trials, it should be made available to anyone who wants to try to use it."* (P30)

© 2026 Forrest R et al. *JAMA Network Open*.
