# Supplementary material for: Patient Perspectives on Approval Speed vs Evidentiary Certainty in US Cancer Drug Approvals
Source: JAMA Netw Open. 2026 Jun 9;9(6):e2617450. doi: 10.1001/jamanetworkopen.2026.17450 (PMC13250706; doi:10.1001/jamanetworkopen.2026.17450)
Supplement: Supplement 2. — Data Sharing Statement [file jamanetwopen-e2617450-s002.pdf]

Data Sharing Statement

Data
Data available: No

Additional Information
Explanation for why data not available: Data are provided in the manuscript or supplementary material.
